# Long-term trends in the burden of leukemia subtypes in China from 1990 to 2021: a Joinpoint regression and age-period-cohort analysis based on GBD 2021

**DOI:** 10.3389/fmed.2026.1826237

**Published:** 2026-06-04

**Authors:** Yudi Shangguan, Xinyue Gou, Yue Luo, Zhuo Chen

**Affiliations:** 1 First Clinical Medical College, Shanxi University of Traditional Chinese Medicine, Jinzhong, China; 2 China Academy of Chinese Medical Sciences, Beijing, China; 3 Department of Hematology, Xiyuan Hospital, China Academy of Chinese Medical Sciences, Beijing, China

**Keywords:** age-period-cohort model, China, disability-adjusted life years, incidence, Joinpoint regression, leukemia subtypes

## Abstract

**Background:**

Leukemia subtypes have distinct epidemiological profiles, but comprehensive comparisons of their long-term burden in China remain limited. This study assessed trends in four major leukemia subtypes from 1990 to 2021 and projected incidence rates over the subsequent 25 years.

**Methods:**

We used data from the Global Burden of Disease (GBD) 2021 database to analyze the burden of acute myeloid leukemia (AML), chronic myeloid leukemia (CML), acute lymphoblastic leukemia (ALL), and chronic lymphocytic leukemia (CLL) in China from 1990 to 2021, stratified by age and sex. Joinpoint regression, age–period–cohort (APC) modeling, and Bayesian APC modeling were applied to assess temporal trends, estimate age, period, and cohort effects, and project future incidence. We also summarized disability-adjusted life years (DALYs) attributable to GBD-estimated risk factors for leukemia and its subtypes.

**Results:**

In 2021, there were an estimated 105,670 new leukemia cases in China. Among the four subtypes, ALL had the highest overall burden, whereas CML had the lowest burden and showed the greatest declines in incidence, deaths, and DALYs. Joinpoint analysis revealed that the age-standardized incidence rates (ASIRs) declined for AML and CML but increased for ALL and CLL. APC analysis indicated that age was the primary determinant of incidence, with distinct age distributions and cohort patterns across subtypes. BAPC projections indicated that incidence rates would remain stable for AML and CML, gradually decline for ALL, and continue to rise for CLL through 2046. Smoking and high body-mass index were the main contributors to attributable DALYs.

**Conclusion:**

Leukemia subtypes in China show distinct long-term incidence trends. The decreasing burden of AML and CML may be associated with improvements in access to diagnosis and treatment, whereas rising ALL and CLL incidence pose new public health challenges, especially among children and the elderly. Tailored, subtype- and age-specific strategies are essential for effective leukemia control and resource planning.

## 1 Introduction

Leukemia is a highly heterogeneous group of hematologic malignancies characterized by the clonal proliferation of abnormal blood cells in the bone marrow and peripheral blood (1, 2). Based on cell lineage and disease progression, it is commonly classified into four main subtypes: acute myeloid leukemia (AML), chronic myeloid leukemia (CML), acute lymphoblastic leukemia (ALL), and chronic lymphocytic leukemia (CLL). These subtypes differ markedly in pathogenesis, epidemiology, risk factors, and clinical outcomes (2). Among them, AML and CLL are the most prevalent in adults, while ALL is the predominant subtype in children (3). Notably, leukemia represents the most prevalent category of childhood malignancies in China (4). Conducting a systematic comparison of long-term trends is essential for identifying subtype-specific patterns and developing targeted prevention and control strategies.

In recent years, the global burden of leukemia, measured by deaths and disability-adjusted life years (DALYs), has shown a decreasing trend across most countries and regions (5). However, leukemia remains a major global health challenge. According to the latest 2022 Global Cancer Observatory (GLOBOCAN) data, leukemia ranked as the 13th most frequently diagnosed cancer and the 10th leading cause of cancer-related mortality, with over 487,000 new cases and 305,000 deaths reported globally (6). Although DALYs may slightly decline, global leukemia cases and deaths are projected to keep rising by 2031 (1). Leukemia incidence shows male predominance (global age-standardized incidence rate [ASIR] ratio: 1.3:1) and peaks between ages 70–74 years (7). Compared with the United States, China has a lower overall leukemia incidence and mortality, but a relatively higher burden of childhood leukemia, highlighting the need for targeted interventions (8). Despite available data, long-term temporal trends of leukemia subtypes in China remain poorly characterized. Current studies on leukemia burden have primarily focused on specific subtypes (9), comparisons between China and the United States (8), urban–rural and gender disparities (10), as well as burden attributable to risk factors (11). However, comprehensive nationwide assessments encompassing all major leukemia subtypes are still lacking.

In this study, the Global Burden of Disease (GBD) 2021 database provides a unique opportunity to assess the long-term trends in incidence, mortality, and DALYs for major leukemia subtypes in China. This study utilizes data spanning from 1990 to 2021, applying Joinpoint regression to detect significant temporal changes, age-period-cohort (APC) models to disentangle the effects of aging, period, and cohort on leukemia risk, and Bayesian APC models to forecast future incidence trends through 2046. The findings aim to offer comprehensive insights into the evolving epidemiology of leukemia subtypes in China and with the ultimate goal of informing evidence-based strategies for prevention, early detection, and resource allocation strategies to effectively reduce disease burden.

## 2 Methods

### 2.1 Data source

This study utilized data from the GBD 2021, which provides comprehensive and standardized estimates for 371 diseases and injuries across 204 countries and territories from 1990 to 2021 (12). We extracted age-standardized rates (ASR) of incidence, deaths, and DALYs for AML, CML, ALL and CLL in China from 1990 to 2021, using the GBD Results Tool^1^. All estimates were stratified by age group, year, and sex. ASRs were calculated using the GBD global standard population, allowing for valid comparisons across populations with different age structures. The GBD framework used statistical models developed by the Institute for Health Metrics and Evaluation (IHME, Seattle, WA, United States), including the Cause of Death Ensemble Model (CODEm) for mortality estimation, Disease Modeling–Meta Regression (DisMod-MR 2.1) for incidence and prevalence estimation, and Spatiotemporal Gaussian Process Regression (ST-GPR) for causes where statistical smoothing across time, age, and location was required. CODEm integrates multiple mortality data sources and applies Bayesian geospatial regression to generate smoothed time trends, while DisMod-MR 2.1 synthesizes diverse epidemiological data to ensure internal consistency and cross-national comparability. ST-GPR enables the modeling of heterogeneous and incomplete datasets by borrowing strength over temporal, spatial, and age dimensions, particularly when DisMod-MR 2.1 cannot adequately model disease parametersv (13, 14).

### 2.2 Statistical analysis

#### 2.2.1 Joinpoint regression analysis

Joinpoint regression analysis is a segmented regression method that identifies significant changes in temporal trends by fitting a series of joined straight lines to time series data (14). We applied Joinpoint regression analysis to assess temporal trends in the ASIR of AML, CML, ALL, and CLL from 1990 to 2021. This method identifies significant time points where a change in the trend occurs. The optimal number of joinpoints was determined by permutation tests with a significance level of 0.05. We calculated the annual percent change and average annual percent change (AAPC), along with their 95% confidence intervals (CIs), for each phase. A statistically significant positive APC indicates an increasing trend, while a negative APC reflects a decreasing trend. All statistical procedures were implemented in R (version 4.4.2).

#### 2.2.2 Age–period–cohort modeling analysis

The APC model is typically formulated as a log-linear model in which the logarithm of incidence rates is expressed as the sum of age, period, and cohort effects (15). In this framework, age, period, and cohort are commonly modeled as categorical variables, and the model includes only main effects without interaction. Age effects reflect variations in leukemia risk across age groups, which may be related to biological aging or accumulated exposures; period effects represent changes affecting the whole population during specific calendar periods, such as improvements in diagnosis, treatment, or public health policy; and cohort effects capture generational differences in risk associated with shared early-life exposures or lifestyle patterns.

However, because age, period, and cohort are exactly linearly dependent, such that cohort = period − age, the APC model suffers from a well-known identification problem (16). To address this issue, we employed the intrinsic estimator (IE) method, which uses singular value decomposition to remove the non-identifiable component and obtain a unique and stable solution without imposing arbitrary conventional constraints (17). The IE method was selected because it directly addresses the non-identifiability of the standard APC model, avoids arbitrary reference-category or constraint-based specifications, and is suitable for estimating age-, period-, and cohort-specific relative risks (RRs) from grouped population-level incidence data. Under this log-linear framework, the estimated coefficients can be interpreted as log-relative risks, which can be exponentiated to obtain RRs for each age group, period, and birth cohort relative to an overall reference level.

In this study, the population was stratified into consecutive 5-year age groups ranging from 0–4 to 95–99 years for AML, CML, and ALL, and from 20–24 to 95–99 years for CLL, in accordance with data availability. The calendar years from 1992 to 2021 were divided into six consecutive 5-year periods (e.g., 1992–1996, 1997–2001, …, 2017–2021), and the corresponding birth cohorts were constructed as overlapping 5-year intervals based on the age–period relationship. A total of 20 age groups, 6 time periods, and 24 birth cohorts were analyzed for AML, CML, and ALL, while 16 age groups, 6 periods, and 21 cohorts were analyzed for CLL due to data unavailability in younger age groups. To support the interpretation and validation of the APC results, we assessed whether the direction and overall pattern of the APC-derived age, period, and cohort effects were broadly consistent with the observed incidence trends and the Joinpoint regression results. Model uncertainty was quantified using 95% confidence intervals for the estimated RRs.

#### 2.2.3 Bayesian age–period–cohort (BAPC) model forecasting

We used the BAPC model to predict the future disease burden of leukemia in China, primarily based on the data structure and research objectives of this study. The incidence of leukemia exhibits a significant age-dependent pattern, and the age distributions of different subtypes vary considerably. Therefore, a simple linear extrapolation based solely on ASRs for the overall population may not adequately reflect the underlying age-specific changes. The BAPC model is based on a generalized linear model within a Bayesian framework and can dynamically integrate age, period, and birth cohort effects (18). These effects are typically assumed to evolve continuously over time and are smoothed using a second-order random walk prior, thereby reducing the impact of random fluctuations on the prediction results while preserving long-term trends (19). Additionally, the BAPC model estimates marginal posterior distributions by integrating nested Laplace approximations. Compared to Markov chain Monte Carlo (MCMC) sampling, INLA replaces the iterative sampling process with a series of numerical approximations, enabling the estimation of marginal posterior distributions and model parameters in latent Gaussian models with high computational efficiency (20). Therefore, we considered the BAPC model suitable for estimating future trends in the burden of leukemia in China.

We implemented the BAPC model using the BAPC R package (version 0.0.36), which relies on Integrated Nested Laplace Approximations (INLA) for computational efficiency. Analyses were conducted in R (version 4.4.2) using RStudio. The BAPC model was fitted using observed incidence data for leukemia subtypes in China from 1990 to 2021, as reported in GBD 2021, and was used to project age-standardized incidence rates over the 25-year period from 2022 to 2046. The year 2046 represents the 25th year after the final observed year, 2021. The analysis was conducted for the total population, with age groups divided into 5-year intervals and both sexes combined. These projections were intended to provide model-based estimates of the future leukemia burden under the assumption that historical trends continue, and should therefore be interpreted as conditional projections rather than precise forecasts.

#### 2.2.4 Risk factors

GBD 2021 adopted a stratified comparative risk assessment framework to evaluate the attributable burden of risk factors, covering 88 risk factors and 631 risk-outcome pairs. For each risk-outcome pair, the GBD estimates the level of risk factor exposure, relative risk, risk-weighted exposure, and theoretical minimum exposure, and uses these to calculate population-attributable scores. These population-attributable scores are then multiplied by the DALYs for the corresponding age, sex, region, and year groups to obtain attributable DALYs (21). This study focuses on the disease burden attributable to four risk factors—high body mass index, occupational benzene exposure, occupational formaldehyde exposure, and smoking—for leukemia and its subtypes in China. We extracted the DALYs, age-standardized DALY rates, and attributable proportions for each risk factor and analyzed them by year, sex, and age group. To describe trends over time, we compared the attributable DALYs and age-standardized DALY rates between 1990 and 2021 and calculated the percentage changes. Additionally, we performed Joinpoint regression analysis on the annual age-standardized DALY rates attributable to each risk factor from 1990 to 2021 to quantify long-term trends.

#### 2.2.5 Uncertainty

Based on the GBD framework, each estimate was generated from 1,000 draws. Point estimates were calculated as the mean across draws, and 95% uncertainty intervals (UIs) were defined by the 25th and 975th ordered values (22). Uncertainty was shown numerically in the text and tables, and graphically as shaded bands in figures that displayed temporal trends or projections.

## 3 Results

### 3.1 Incidence of leukemia and their trends in China, 1990–2021

**Figure 1:**
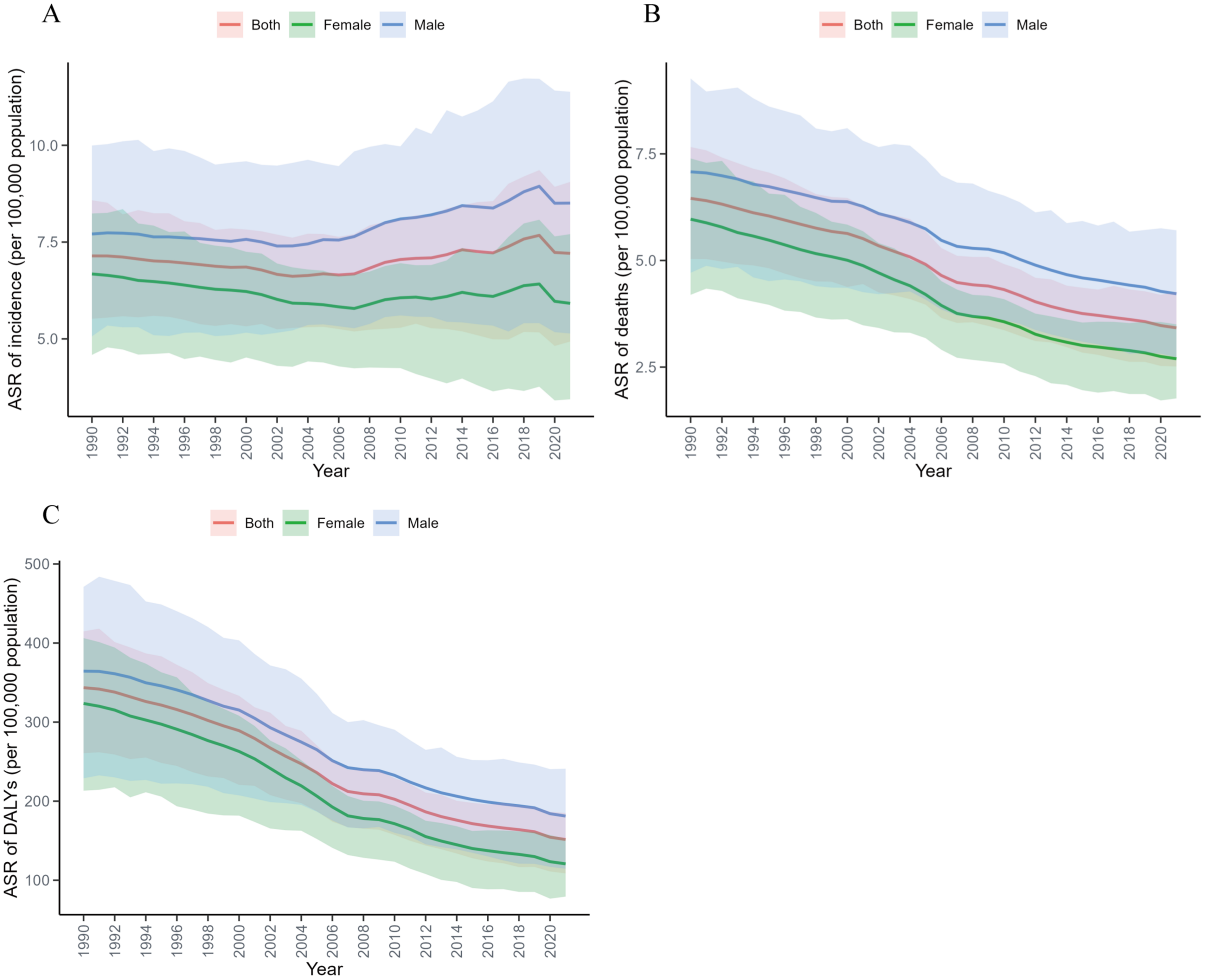
Trends in age-standardized rates of incidence, death, and DALYs for leukemia in China, 1990–2021 **(A)** incidence, **(B)** death, and **(C)** DALYs per 100,000 population. Shaded areas indicate 95% uncertainty intervals. Results are shown for both sexes, males, and females. Data source: Global Burden of Disease Study 2021. DALYs, disability-adjusted life years; UIs, uncertainty intervals.

Between 1990 and 2021, the incidence of leukemia in China remained essentially stable, with no significant long-term variation. During this period, the ASIR changed only slightly from 7.14 (95% uncertainty interval [UI]: 5.52, 8.58) to 7.21 (95% UI: 4.93, 9.05) per 100,000 population (Table 1 and Figure 1A). Consistently, the AAPC indicated a non-significant decline of −0.01% (95% CI: −0.10 to 0.05; *p* = 0.8) (Table 2). Among the subtypes, CML showed the most notable decrease, with an annual decline of 1.85% (95% CI: −1.91, −1.78), and the ASIR fell from 0.38 (95% UI: 0.17, 0.55) to 0.21 (95% UI: 0.11, 0.33) per 100,000 population. AML also showed a decline, with an annual decrease of 1.10% (95% CI: −1.13, −1.06), with ASIR falling from 1.46 (95% UI: 0.80, 2.24) to 1.03 (95% UI: 0.69, 1.45) (Tables 1, 2 and Supplementary Figures S1A,B).

**Table 1:** The absolute number and age-standardized rates of incidence, deaths, and DALYs for leukemia and its subtypes in China, 1990–2021.

Cause	Measure	Sex	Absolute numbers (thousands)			Age-standardized rate (per 100,000)		
			1990	2021	Percentage change, 1990–2021 (%)	1990	2021	Percentage change, 1990–2021 (%)
Leukemia	DALYs	Both	3924.47(2969.93, 4726.96)	2205.22(1612.84, 2736.63)	−0.44(−0.55, −0.26)	343.57(260.79, 414.78)	151.54(108.71, 185.06)	−0.56(−0.65, −0.42)
Leukemia	DALYs	Female	1771.36(1167.26, 2217.58)	871.24(560.29, 1123.71)	−0.51(−0.65, −0.29)	323.54(213.29, 406.25)	120.73(79.29, 152.53)	−0.63(−0.73, −0.45)
Leukemia	DALYs	Male	2153.11(1344.3, 2780.82)	1333.98(851.89, 1808.56)	−0.38(−0.55, 0.03)	364.46(229.02, 471.03)	181.16(114.56, 241.04)	−0.5(−0.64, −0.19)
Leukemia	Deaths	Both	67.42(52.04, 80.04)	58.9(43.63, 74.04)	−0.13(−0.29, 0.15)	6.46(5.04, 7.66)	3.42(2.51, 4.26)	−0.47(−0.57, −0.31)
Leukemia	Deaths	Female	30.68(21.23, 38.19)	23.74(15.47, 31.18)	−0.23(−0.44, 0.08)	5.97(4.19, 7.39)	2.7(1.77, 3.5)	−0.55(−0.67, −0.37)
Leukemia	Deaths	Male	36.74(24.16, 47.47)	35.17(22.77, 47.96)	−0.04(−0.31, 0.47)	7.08(4.71, 9.27)	4.22(2.73, 5.71)	−0.4(−0.56, −0.12)
Leukemia	Incidence	Both	76.2(58.31, 90.96)	105.67(75.28, 132.24)	0.39(0.08, 0.83)	7.14(5.52, 8.58)	7.21(4.93, 9.05)	0.01(−0.23, 0.35)
Leukemia	Incidence	Female	34.76(23.69, 42.94)	43.1(26.05, 55.83)	0.24(−0.17, 0.75)	6.68(4.59, 8.24)	5.92(3.44, 7.7)	−0.11(−0.43, 0.26)
Leukemia	Incidence	Male	41.44(27.23, 53.53)	62.56(38.66, 84.01)	0.51(0.07, 1.43)	7.71(5.07, 9.99)	8.51(5.14, 11.38)	0.1(−0.23, 0.77)
AML	DALYs	Both	851.93(419.99, 1454.75)	548.56(373.86, 778.26)	−0.36(−0.65, 0.18)	75.09(37.56, 127.44)	36.96(25.32, 52.46)	−0.51(−0.73, −0.11)
AML	DALYs	Female	370.24(159.64, 601.66)	234.89(154.26, 386.16)	−0.37(−0.66, 0.52)	68.02(29.79, 110.01)	32.12(21.15, 52.66)	−0.53(−0.75, 0.16)
AML	DALYs	Male	481.7(174.26, 896.33)	313.67(160.12, 478.14)	−0.35(−0.69, 0.22)	82.09(30.52, 151.23)	41.75(21.41, 64.13)	−0.49(−0.76, −0.08)
AML	Deaths	Both	14.85(8.01, 23.4)	15.31(10.37, 21.4)	0.03(−0.39, 0.79)	1.45(0.8, 2.21)	0.88(0.59, 1.24)	−0.4(−0.63, 0.01)
AML	Deaths	Female	6.64(3.47, 10.08)	6.62(4.26, 10.83)	0(−0.42, 1.03)	1.31(0.69, 1.94)	0.74(0.49, 1.22)	−0.43(−0.67, 0.14)
AML	Deaths	Male	8.21(3.24, 14.25)	8.69(4.56, 13.26)	0.06(−0.44, 0.78)	1.62(0.7, 2.67)	1.03(0.55, 1.59)	−0.36(−0.64, 0.04)
AML	Incidence	Both	15.31(8.21, 24.14)	17.84(11.88, 24.8)	0.16(−0.31, 1.01)	1.46(0.8, 2.24)	1.03(0.69, 1.45)	−0.3(−0.57, 0.15)
AML	Incidence	Female	6.86(3.56, 10.37)	7.96(5.01, 13)	0.16(−0.33, 1.37)	1.34(0.7, 1.99)	0.91(0.59, 1.47)	−0.32(−0.61, 0.37)
AML	Incidence	Male	8.45(3.35, 14.82)	9.88(5.05, 15.04)	0.17(−0.39, 0.92)	1.61(0.69, 2.71)	1.17(0.6, 1.78)	−0.28(−0.6, 0.16)
CML	DALYs	Both	177.8(80.35, 260.19)	60.93(35.08, 101.13)	−0.66(−0.79, −0.35)	15.59(7.13, 22.7)	3.65(2.06, 6.03)	−0.77(−0.86, −0.56)
CML	DALYs	Female	82.96(30.5, 138.67)	25.16(15.93, 42.35)	−0.7(−0.84, −0.28)	15.05(5.63, 25.14)	2.97(1.86, 5.21)	−0.8(−0.9, −0.55)
CML	DALYs	Male	94.83(22.16, 139.05)	35.77(8.93, 64.35)	−0.62(−0.77, −0.25)	16.15(3.82, 23.68)	4.33(1.07, 8)	−0.73(−0.84, −0.5)
CML	Deaths	Both	3.59(1.69, 5.24)	1.84(1.08, 3.09)	−0.49(−0.69, −0.04)	0.36(0.17, 0.52)	0.1(0.06, 0.16)	−0.72(−0.83, −0.5)
CML	Deaths	Female	1.75(0.71, 2.92)	0.79(0.5, 1.36)	−0.55(−0.76, 0.05)	0.35(0.15, 0.58)	0.08(0.05, 0.14)	−0.77(−0.88, −0.5)
CML	Deaths	Male	1.85(0.42, 2.79)	1.05(0.26, 1.89)	−0.43(−0.65, 0.07)	0.37(0.08, 0.57)	0.12(0.03, 0.22)	−0.67(−0.79, −0.41)
CML	Incidence	Both	3.92(1.82, 5.75)	3.85(2.09, 6.11)	−0.02(−0.39, 0.77)	0.38(0.17, 0.55)	0.21(0.11, 0.33)	−0.44(−0.65, −0.03)
CML	Incidence	Female	1.89(0.76, 3.15)	1.66(0.98, 2.64)	−0.12(−0.53, 0.84)	0.37(0.15, 0.62)	0.18(0.1, 0.28)	−0.52(−0.74, −0.03)
CML	Incidence	Male	2.02(0.47, 3.05)	2.19(0.51, 3.91)	0.08(−0.34, 0.93)	0.39(0.09, 0.59)	0.25(0.06, 0.44)	−0.36(−0.59, 0.11)
ALL	DALYs	Both	2275.29(1587.18, 3041.61)	924.42(525.9, 1182.32)	−0.59(−0.75, −0.38)	195.79(136.54, 262.37)	74.06(42.86, 94.99)	−0.62(−0.77, −0.43)
ALL	DALYs	Female	1062.64(656.91, 1493.84)	352.16(150.15, 466.73)	−0.67(−0.85, −0.5)	192.21(118.57, 271.45)	57.68(26.26, 77.18)	−0.7(−0.86, −0.53)
ALL	DALYs	Male	1212.65(604.4, 1778.23)	572.26(271.05, 806.39)	−0.53(−0.74, −0.01)	199.89(99.32, 294.88)	89.07(43.59, 123.31)	−0.55(−0.75, −0.06)
ALL	Deaths	Both	33.92(23.96, 44.73)	20.61(11.78, 27.3)	−0.39(−0.62, −0.07)	3.05(2.17, 4.02)	1.36(0.78, 1.75)	−0.56(−0.72, −0.34)
ALL	Deaths	Female	15.91(9.81, 21.98)	8.13(3.27, 11.27)	−0.49(−0.76, −0.22)	2.97(1.81, 4.12)	1.06(0.45, 1.4)	−0.64(−0.83, −0.46)
ALL	Deaths	Male	18.01(9.65, 25.9)	12.48(5.59, 18.05)	−0.31(−0.61, 0.37)	3.16(1.75, 4.64)	1.65(0.77, 2.33)	−0.48(−0.7, 0.01)
ALL	Incidence	Both	38.03(26.76, 50.35)	38.57(21.15, 50.76)	0.01(−0.41, 0.54)	3.38(2.39, 4.49)	3.64(2, 5.05)	0.07(−0.4, 0.67)
ALL	Incidence	Female	18.09(11.1, 25.06)	15.39(6.37, 21.16)	−0.15(−0.63, 0.35)	3.36(2.03, 4.69)	3.03(1.29, 4.37)	−0.1(−0.63, 0.56)
ALL	Incidence	Male	19.94(10.36, 29.12)	23.18(10.99, 32.71)	0.16(−0.37, 1.53)	3.44(1.85, 5.03)	4.2(1.92, 6.08)	0.22(−0.36, 1.74)
CLL	DALYs	Both	176.96(101.44, 240.86)	268.25(165.77, 380.36)	0.52(0.11, 1.17)	17.07(9.95, 23.04)	14.11(8.74, 19.93)	−0.17(−0.39, 0.16)
CLL	DALYs	Female	74.54(33.29, 118.35)	98.3(40.95, 145.25)	0.32(−0.26, 1.66)	14.52(6.81, 22.4)	10.16(4.18, 15.17)	−0.3(−0.6, 0.36)
CLL	DALYs	Male	102.42(43.78, 146.4)	169.95(89.5, 260.96)	0.66(0.07, 1.75)	19.9(8.8, 28.2)	18.26(9.52, 27.62)	−0.08(−0.41, 0.47)
CLL	Deaths	Both	4.85(2.82, 6.57)	8.64(5.53, 12.38)	0.78(0.33, 1.49)	0.55(0.33, 0.74)	0.44(0.28, 0.63)	−0.2(−0.4, 0.11)
CLL	Deaths	Female	2.05(0.99, 3.14)	3.15(1.33, 4.6)	0.53(−0.1, 1.88)	0.45(0.23, 0.66)	0.31(0.13, 0.45)	−0.32(−0.59, 0.24)
CLL	Deaths	Male	2.8(1.27, 4)	5.49(2.95, 8.59)	0.96(0.26, 2.16)	0.68(0.33, 0.97)	0.6(0.33, 0.93)	−0.12(−0.42, 0.38)
CLL	Incidence	Both	6.78(3.99, 9.16)	28.93(17.96, 40.58)	3.27(2.15, 5.04)	0.72(0.42, 0.98)	1.42(0.88, 2)	0.97(0.48, 1.76)
CLL	Incidence	Female	3.03(1.46, 4.68)	12.09(5.03, 17.52)	2.98(1.31, 6.61)	0.63(0.32, 0.95)	1.15(0.48, 1.66)	0.82(0.07, 2.43)
CLL	Incidence	Male	3.75(1.67, 5.43)	16.84(8.39, 26.71)	3.49(1.89, 6.43)	0.83(0.39, 1.2)	1.71(0.87, 2.69)	1.06(0.35, 2.28)

Data are presented as absolute numbers (in thousands) and age-standardized rates (per 100,000 population), with 95% uncertainty intervals (UIs) in parentheses. Percentage change refers to the change between 1990 and 2021. DALYs, disability-adjusted life years; AML, acute myeloid leukemia; CML, chronic myeloid leukemia; ALL, acute lymphoblastic leukemia; CLL, chronic lymphocytic leukemia. Data source: Global Burden of Disease Study 2021.

**Table 2:** Trends in age-standardized incidence, death, and DALY rates of leukemia subtypes in China, 1990–2021.

Measure	Sex	Leukemia		AML		CML		ALL		CLL	
		AAPC	*P*	AAPC	*P*	AAPC	*P*	AAPC	*P*	AAPC	*P*
ASR of incidence	Both	−0.01 (−0.10, 0.05)	0.8	−1.10 (−1.13, −1.06)	<0.001	−1.85 (−1.91, −1.78)	<0.001	0.15 (0.03, 0.26)	0.01	2.25 (2.20, 2.30)	<0.001
	Female	−0.44 (−0.51, −0.38)	<0.001	−1.22 (−1.26, −1.17)	<0.001	−2.33 (−2.42, −2.25)	<0.001	−0.42 (−0.55, −0.30)	<0.001	1.98 (1.92, 2.04)	<0.001
	Male	0.27 (0.19, 0.33)	<0.001	−1.04 (−1.06, −1.00)	<0.001	−1.43 (−1.51, −1.36)	<0.001	0.57 (0.46, 0.67)	<0.001	2.39 (2.34, 2.44)	<0.001
ASR of deaths	Both	−2.03 (−2.05, −2.01)	<0.001	−1.60 (−1.62, −1.56)	<0.001	−4.04 (−4.09, −3.99)	<0.001	−2.59 (−2.64, −2.55)	<0.001	−0.71 (−0.74, −0.69)	<0.001
	Female	−2.53 (−2.55, −2.50)	<0.001	−1.77 (−1.82, −1.72)	<0.001	−4.58 (−4.63, −4.53)	<0.001	−3.29 (−3.34, −3.24)	<0.001	−1.22 (−1.26, −1.18)	<0.001
	Male	−1.67 (−1.69, −1.65)	<0.001	−1.46 (−1.49, −1.43)	<0.001	−3.57 (−3.65, −3.48)	<0.001	−2.08 (−2.12, −2.03)	<0.001	−0.43 (−0.47, −0.38)	<0.001
ASR of DALYs	Both	−2.61 (−2.65, −2.56)	<0.001	−2.27 (−2.32, −2.22)	<0.001	−4.61 (−4.66, −4.56)	<0.001	−3.12 (−3.18, −3.05)	<0.001	−0.60 (−0.63, −0.56)	<0.001
	Female	−3.15 (−3.19, −3.10)	<0.001	−2.35 (−2.39, −2.31)	<0.001	−5.13 (−5.19, −5.06)	<0.001	−3.87 (−3.92, −3.82)	<0.001	−1.13 (−1.18, −1.07)	<0.001
	Male	−2.24 (−2.30, −2.19)	<0.001	−2.19 (−2.25, −2.13)	<0.001	−4.21 (−4.28, −4.15)	<0.001	−2.58 (−2.64, −2.52)	<0.001	−0.27 (−0.29, −0.24)	<0.001

Data are presented as average annual percent change (AAPC) with 95% confidence intervals (CIs) and corresponding p-values. DALYs, disability-adjusted life years; AML, acute myeloid leukemia; CML, chronic myeloid leukemia; ALL, acute lymphoblastic leukemia; CLL, chronic lymphocytic leukemia.

Notably, from 1990 to 2021, two leukemia subtypes showed a substantial increase in incidence. CLL showed the most notable increase, with an AAPC of 2.25% (95% CI: 2.20, 2.30), as the ASIR rose from 0.72 (95% UI: 0.42, 0.98) to 1.42 (95% UI: 0.88, 2.00). ALL exhibited a modest increase of 0.15% per year (95% CI: 0.03, 0.26), with ASIR increasing from 3.38 (95% UI: 2.39, 4.49) to 3.64 (95% UI: 2.00, 5.05) per 100,000 population (Tables 1, 2 and Supplementary Figures S1C,D).

**Figure 2:**
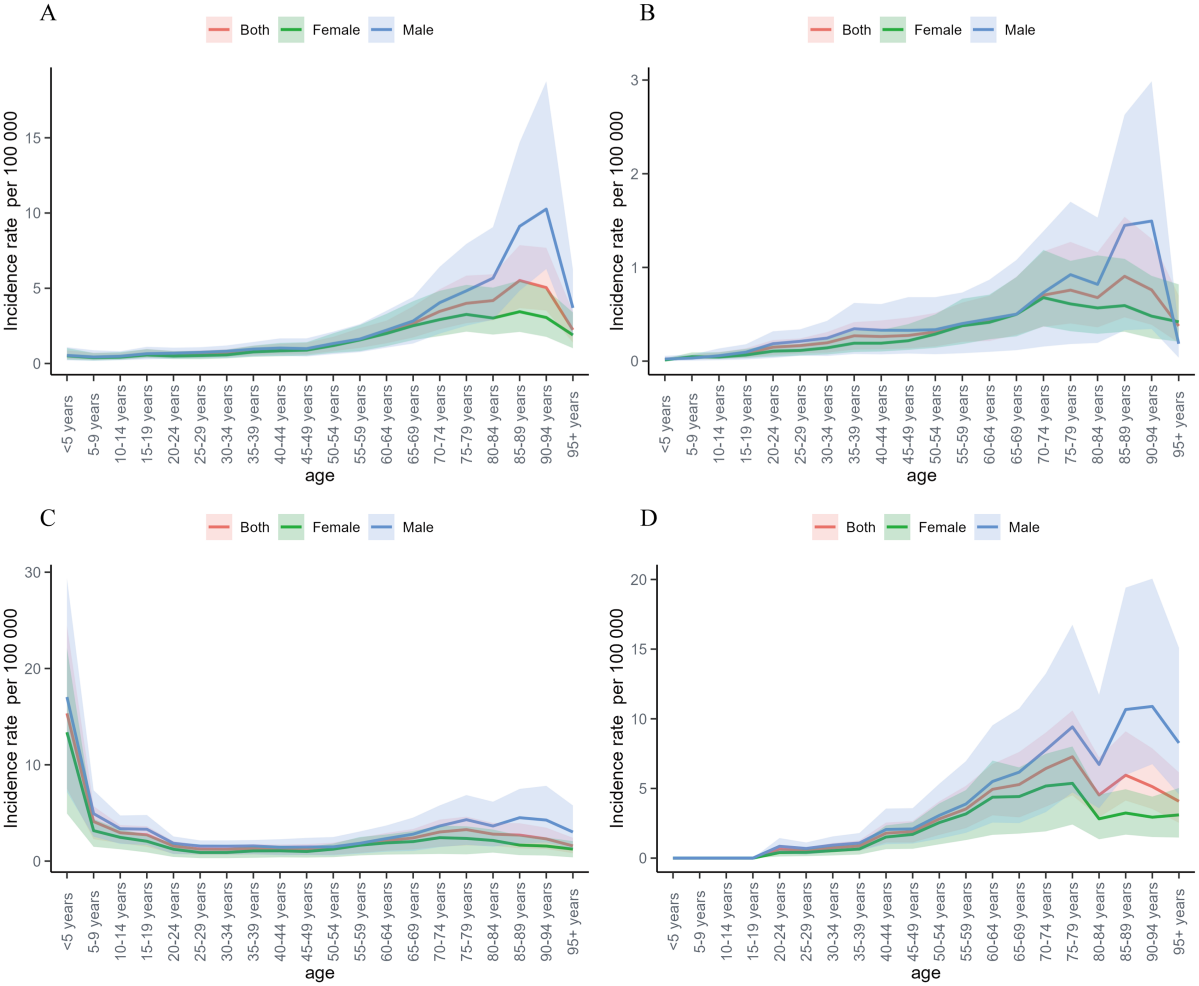
Age-specific incidence rates of leukemia subtypes in China by sex, 2021 **(A)** AML, **(B)** CML, **(C)** ALL, **(D)** CLL. Incidence rates are expressed per 100,000 population and stratified by sex (both sexes, males, and females). Shaded areas represent 95% uncertainty intervals. Data source: Global Burden of Disease Study 2021. AML, acute myeloid leukemia; CML, chronic myeloid leukemia; ALL, acute lymphoblastic leukemia; CLL, chronic lymphocytic leukemia.

In 2021, the highest ASIR of leukemia in China was observed in ALL, with a rate of 3.64 (95% UI: 2.00, 5.05) per 100,000 population, followed by CLL with an ASIR of 1.42 (95% UI: 0.88, 2.00), AML with an ASIR of 1.03 (95% UI: 0.69, 1.45), and CML with an ASIR of 0.21 (95% UI: 0.11, 0.33) (Table 1). The age and sex distribution of leukemia incidence in 2021 revealed distinct patterns across subtypes. ALL incidence was highest in individuals aged <50 years, particularly among children and young adults. In contrast, CLL incidence predominantly occurred in adults aged ≥50 years. For AML and CML, incidence rates steadily increased with age, with the ASIR peaking in the 85–89 years age group. Across all subtypes, males generally had higher incidence rates than females, with more pronounced differences in older age groups for CML and CLL (Supplementary Table S1 and Figure 2).

### 3.2 Deaths of leukemia and their trends in China, 1990–2021

From 1990 to 2021, the age-standardized death rate (ASDR) of leukemia in China decreased from 6.46 (95% UI: 5.04, 7.66) to 3.42 (95% UI: 2.52, 4.26) per 100,000 population, with an average annual decline of 2.03% (95% CI: −2.05, −2.01) (Tables 1, 2 and Figure 1B). All four major leukemia subtypes exhibited a decreasing trend in ASDR during this period (Supplementary Figure S2). CML exhibited the fastest decline, with an AAPC of −4.04% (95% CI: −4.09, −3.99), and its ASDR decreased from 0.36 (95% UI: 0.17, 0.52) to 0.10 (95% UI: 0.06, 0.16) per 100,000 population. This was followed by ALL, which showed a substantial reduction in ASDR from 3.05 (95% UI: 2.17, 4.02) to 1.36 (95% UI: 0.78, 1.75), with an AAPC of −2.59% (95% CI: −2.64, −2.55). AML also showed a decline, with ASDR falling from 1.45 (95% UI: 0.80, 2.21) to 0.88 (95% UI: 0.59, 1.24), and an AAPC of −1.60% (95% CI: −1.62, −1.56). In contrast, CLL experienced the slowest decline, with rates decreasing from 0.55 (95% UI: 0.33, 0.74) to 0.44 (95% UI: 0.28, 0.63), and an AAPC of −0.71% (95% CI: −0.74, −0.69) (Tables 1, 2 and Supplementary Figure S2).

**Figure 3:**
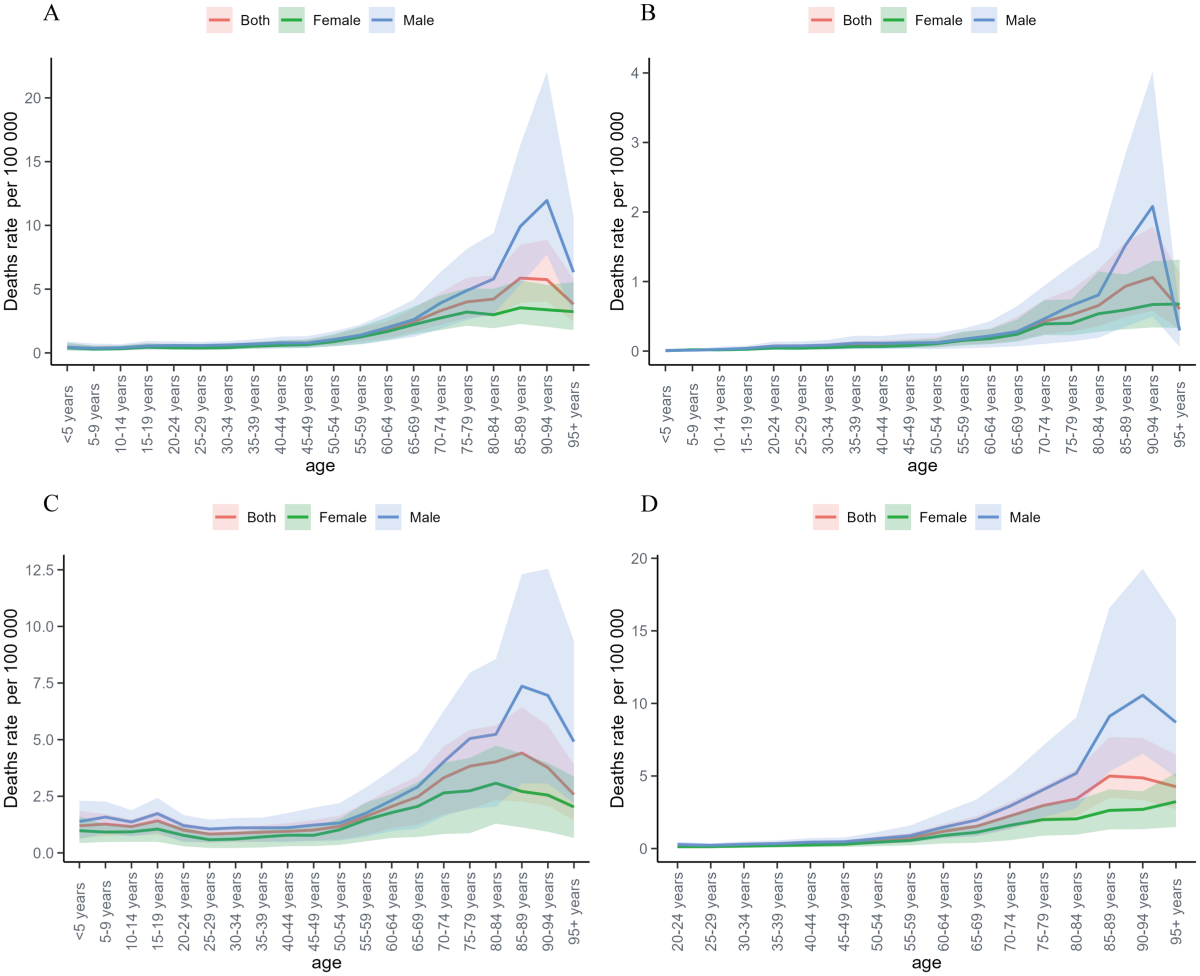
Age-specific death rates of leukemia subtypes in China by sex, 2021 **(A)** AML, **(B)** CML, **(C)** ALL, **(D)** CLL. Death rates are expressed per 100,000 population and stratified by sex (both sexes, males, and females). Shaded areas indicate 95% uncertainty intervals. Data source: Global Burden of Disease Study 2021. AML, acute myeloid leukemia; CML, chronic myeloid leukemia; ALL, acute lymphoblastic leukemia; CLL, chronic lymphocytic leukemia.

In 2021, the highest ASDR was observed for ALL (1.36 [95% UI: 0.78, 1.75]), followed by AML (0.88 [95% UI: 0.59, 1.24]), CLL (0.44 [95% UI: 0.28, 0.63]) and CML (0.10 [95% UI: 0.06, 0.16]) (Table 1). Among the subtypes, ALL showed the highest mortality rates and death counts predominantly in individuals aged <70 years. In contrast, AML mortality peaked in the population aged ≥70 years, surpassing other subtypes in this age group. Mortality rates for CML and CLL steadily increased with age. For all four leukemia subtypes, the highest mortality rates were observed in the oldest age group, those aged ≥85 years. Overall, males consistently exhibited higher mortality rates than females (Supplementary Table S1 and Figure 3).

### 3.3 DALYs of leukemia and their trends in China, 1990–2021

From 1990 to 2021, the age-standardized DALY rate of leukemia in China decreased from 343.57 (95% UI: 260.79, 414.78) to 151.54 (95% UI: 108.71, 185.06) per 100,000 population, with an average annual decline of 2.61% (95% CI: −2.65, −2.56) (Tables 1, 2 and Figure 1C). All four major leukemia subtypes showed a decreasing trend in DALY rates during this period (Supplementary Figure S3). Among them, CML exhibited the largest average annual percentage decline, decreasing by 4.61% per year (95% CI: −4.66, −4.56), with rates falling from 15.59 to 3.65 per 100,000 population. This was followed by ALL, whose age-standardized DALY rate declined from 195.79 (95% UI: 136.54, 262.37) to 74.06 (95% UI: 42.86, 94.99) per 100,000 population, with an AAPC of −3.12% (95% CI: −3.18, −3.05). AML also showed a notable reduction, with its DALY rate decreasing from 75.09 (95% UI: 37.56, 127.44) to 36.96 (95% UI: 25.32, 52.46) per 100,000 population (AAPC: −2.27 [95% CI: −2.32, −2.22]). In contrast, CLL experienced the smallest decline, with a rate drop from 17.07 (95% UI: 9.95, 23.04) to 14.11 (95% UI: 8.74, 19.93) per 100,000 population and an AAPC of −0.60% (95% CI: −0.63, −0.56) (Tables 1, 2 and Supplementary Figure S3).

**Figure 4:**
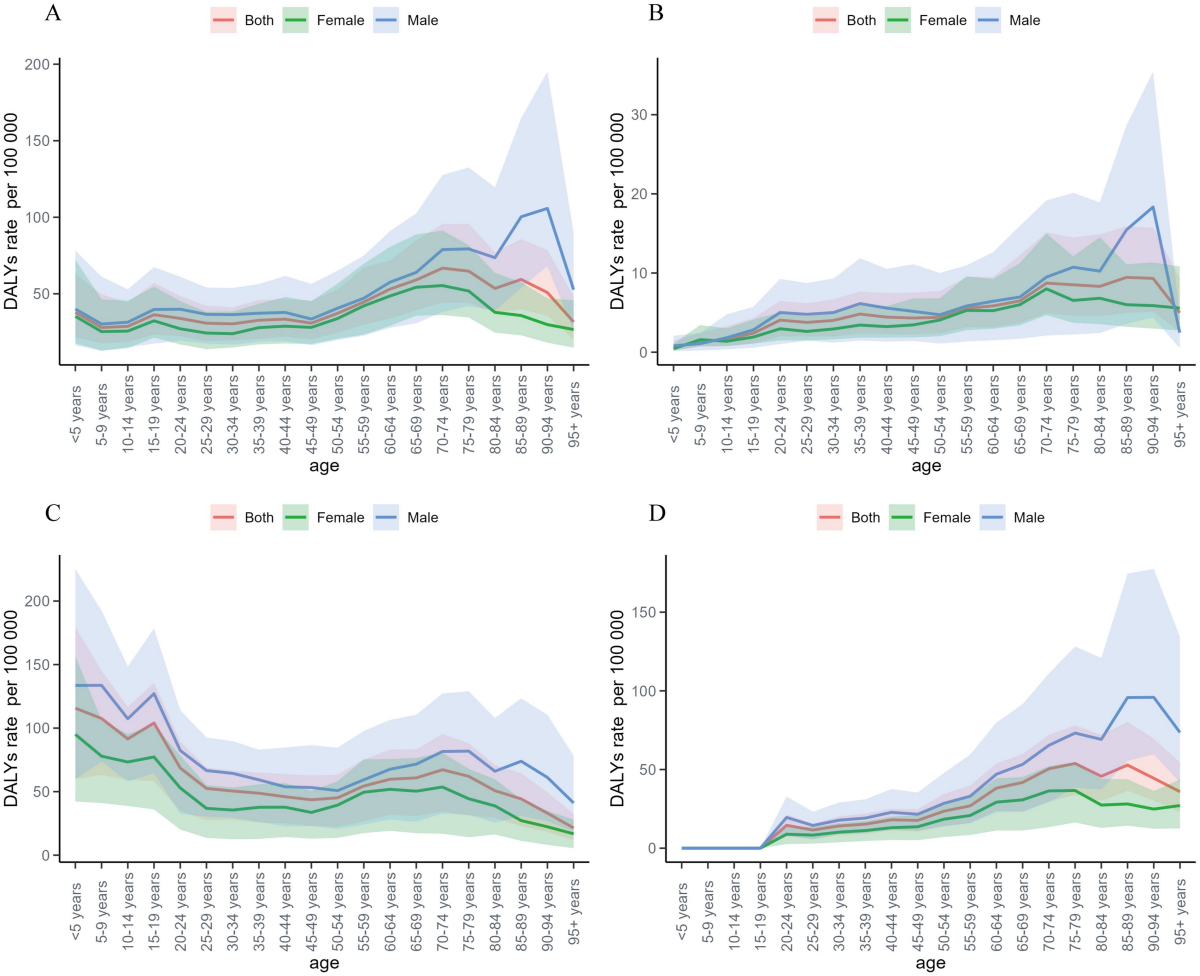
Age-specific DALY rates of leukemia subtypes in China by sex, 2021: **(A)** AML; **(B)** CML; **(C)** ALL; **(D)** CLL. DALY rates are expressed per 100,000 population and stratified by sex (both sexes, males, and females). Shaded areas represent 95% uncertainty intervals. Data source: Global Burden of Disease Study 2021. DALYs, disability-adjusted life years; AML, acute myeloid leukemia; CML, chronic myeloid leukemia; ALL, acute lymphoblastic leukemia; CLL, chronic lymphocytic leukemia.

In 2021, among leukemia subtypes in China, ALL accounted for the highest DALYs per 100,000 population at 74.06 (95% UI: 42.86, 94.99), followed by AML at 36.96 (95% UI: 25.32, 52.46), CLL at 14.11 (95% UI: 8.74, 19.93), and CML at 3.65 (95% UI: 2.06, 6.03). Males exhibited significantly higher total DALYs and ASRs across all subtypes compared to females (Table 1). Age-stratified analysis revealed that ALL burden was predominantly concentrated in individuals aged <70 years, with peak DALYs observed in early childhood, particularly in the <5 years and 5–9 years age groups. Conversely, AML burden peaked in those aged ≥70 years. For CML and CLL, disease burden increased progressively with age (Figure 4 and Supplementary Table S1).

### 3.4 Joinpoint regression analysis of AML, CML, ALL, and CLL incidence trends

**Figure 5:**
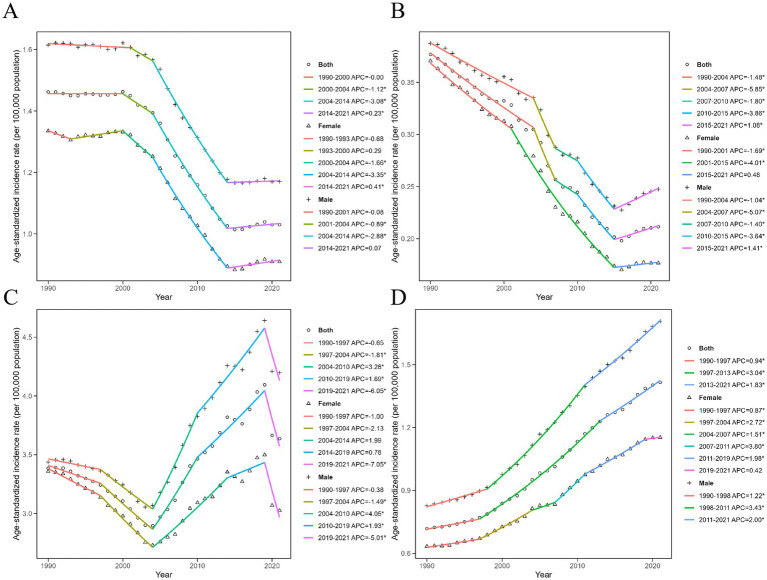
Joinpoint regression analysis of age-standardized incidence rates for leukemia subtypes in China, 1990–2021: **(A)** AML; **(B)** CML; **(C)** ALL; **(D)** CLL. Age-standardized incidence rates are expressed per 100,000 population and are shown for both sexes combined, females, and males. Lines indicate segmented APCs (annual percent changes); * denotes statistical significance (*p* < 0.05). Data source: Global Burden of Disease Study 2021. AML, acute myeloid leukemia; CML, chronic myeloid leukemia; ALL, acute lymphoblastic leukemia; CLL, chronic lymphocytic leukemia; APC, annual percent change.

The Joinpoint regression analysis revealed distinct temporal patterns in the ASIRs of AML, CML, ALL, and CLL in China from 1990 to 2021 (Figure 5). AML and CML both demonstrated long-term downward trends, followed by modest recent increases. For CML, the sharpest decline occurred between 2004 and 2007, but since 2015, a significant upward shift has emerged, especially among males, whereas females maintained an overall declining trajectory. AML showed a similar pattern, with a marked reduction between 2000 and 2014, particularly from 2004 to 2014, followed by a slight rebound after 2014, more evident in females (Figures 5A,B and Supplementary Tables S2, S3).

In contrast, ALL and CLL exhibited rising trends. The ASIR of CLL increased steadily throughout the study period, accelerating between 1997 and 2013 and continuing at a slower but still significant pace thereafter, with males experiencing steeper growth than females. ALL incidence fluctuated, declining slightly before 2004, then rising rapidly between 2004 and 2010 and moderately until 2019. From 2019 to 2021, ALL incidence declined sharply (Figures 5C,D and Supplementary Tables S4, S5).

### 3.5 Age, period, and cohort effects on the incidence of AML, CML, ALL, and CLL

**Figure 6:**
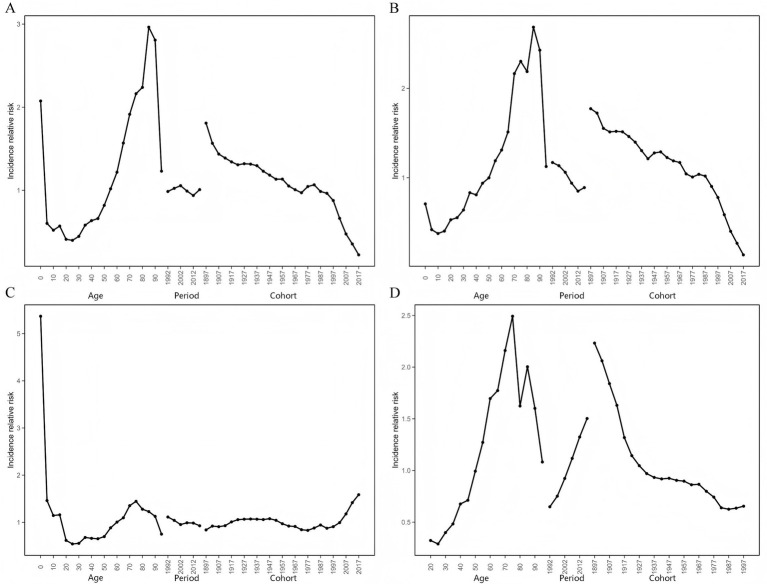
Age, period, and cohort effects on the incidence of leukemia subtypes in China, 1990–2021 **(A)** AML; **(B)** CML; **(C)** ALL; **(D)** CLL. Relative risks (RRs) were estimated using the age–period–cohort model. The *y*-axis represents incidence relative risk, while the *x*-axis denotes age (left), period (middle), and birth cohort (right). RR, relative risk; AML, acute myeloid leukemia; CML, chronic myeloid leukemia; ALL, acute lymphoblastic leukemia; CLL, chronic lymphocytic leukemia.

The APC analysis further revealed long-term patterns in the incidence of AML, CML, ALL, and CLL in China. Among the three components, age effect consistently dominated the variation in incidence rates across all leukemia subtypes, showing substantial differences in relative risks (RRs) between age groups. ALL displayed a distinctive childhood surge, with the relative risk (RR) peaking sharply at ages 0–4 at 5.37 (95% CI: 5.04, 5.72), followed by a pronounced decline in young adulthood and a modest rebound in later life. By contrast, AML, CML, and CLL demonstrated progressive, monotonic increases with advancing age, with the highest risks observed in the oldest groups: AML reached 2.96 (95% CI: 2.83, 3.10) and CML reached 2.68 (95% CI: 2.44, 2.96) at ages 85–89, while CLL attained its maximum of 2.49 (95% CI: 2.42, 2.57) at ages 75–79 (Figure 6 and Supplementary Table S6).

The period effect analysis demonstrated a decreasing trend in RR over time for AML, CML, and ALL. In contrast, CLL exhibited an increasing period effect across the study period. For AML, the period RRs remained generally stable across the entire period from 1992 to 2021, fluctuating narrowly between 0.94 and 1.06. For CML, a significant downward trend was observed, with RRs declining from 1.17 (95% CI: 1.14, 1.20) to 0.89 (95% CI: 0.86, 0.92), indicating a steady reduction in period-associated risk. In contrast, ALL showed a gradual decrease in period RRs from 1.11 (95% CI: 1.09, 1.13) to 0.93 (95% CI: 0.91, 0.94) over the same time frame. Notably, CLL demonstrated a marked and consistent increase, with RRs rising from 0.65 (95% CI: 0.64, 0.66) to 1.50 (95% CI: 1.47, 1.53), suggesting substantial period-related increases in risk (Figure 6 and Supplementary Table S6).

Cohort effects further underscored generational differences. AML, CML, and CLL all demonstrated progressive declines across successive birth cohorts, consistent with long-term improvements in population-level risk exposures. In contrast, ALL showed a marked cohort effect, with risks increasing steadily among cohorts born after 2007. Taken together, these results highlight that age effects dominated across all subtypes, whereas period and cohort effects revealed heterogeneous trajectories, most notably the post-2007 increase in ALL cohorts and the persistent rise in CLL across periods (Figure 6 and Supplementary Table S6).

### 3.6 Projected trends in the burden of AML, CML, ALL, and CLL in China through 2046

**Figure 7:**
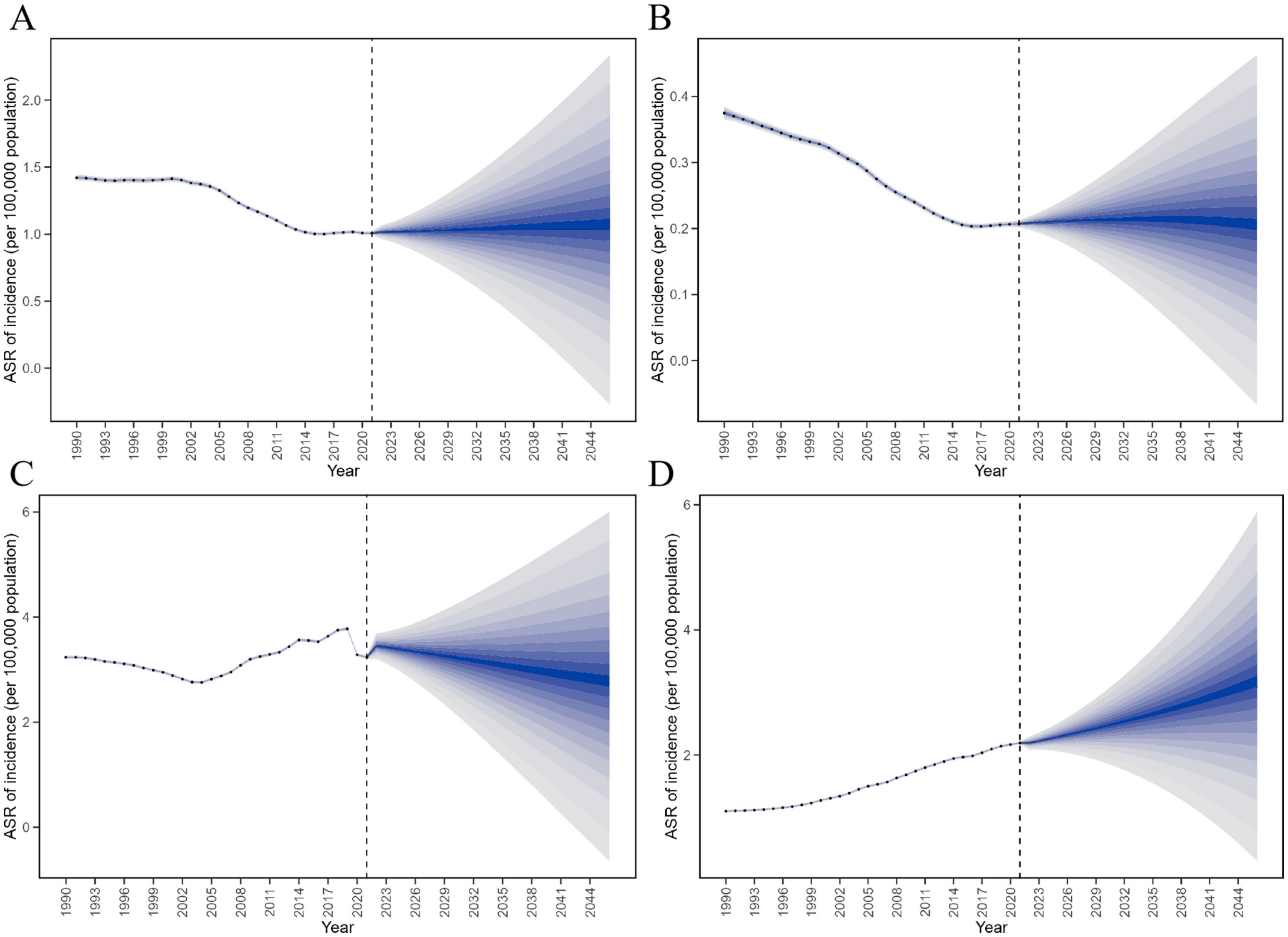
BAPC model projections of age-standardized incidence rates for leukemia subtypes in China among both sexes combined, 1990–2046: **(A)** AML; **(B)** CML; **(C)** ALL; **(D)** CLL. Dashed lines indicate the separation between observed data (1990–2021) and projected data (2022–2046). Shaded areas represent 95% uncertainty intervals. Data source: Global Burden of Disease Study 2021. ASR, age-standardized rate; AML, acute myeloid leukemia; CML, chronic myeloid leukemia; ALL, acute lymphoblastic leukemia; CLL, chronic lymphocytic leukemia; BAPC, Bayesian age–period–cohort.

It is projected that from 2022 to 2046, the ASIRs of the four leukemia subtypes in China will show heterogeneous patterns, with all rates expressed per 100,000 population. AML and CML will show only slight changes in ASIR, with AML expected to increase marginally from 1.01 to 1.03 and CML expected to decrease slightly from 0.21 to 0.20. In contrast, ALL and CLL will exhibit more pronounced divergent trends, with ALL projected to decline from 3.44 to 2.68 and CLL projected to increase from 2.19 to 3.10. In terms of absolute case numbers, AML, CML, and CLL are expected to continue increasing, which may partly reflect the influence of population growth and aging. Only ALL is expected to decrease in both ASIR and incident case numbers (Figure 7; Supplementary Figure S4; Supplementary Table S7).

### 3.7 DALYs from leukemia and its subtypes attributable to selected risk factors

**Figure 8:**
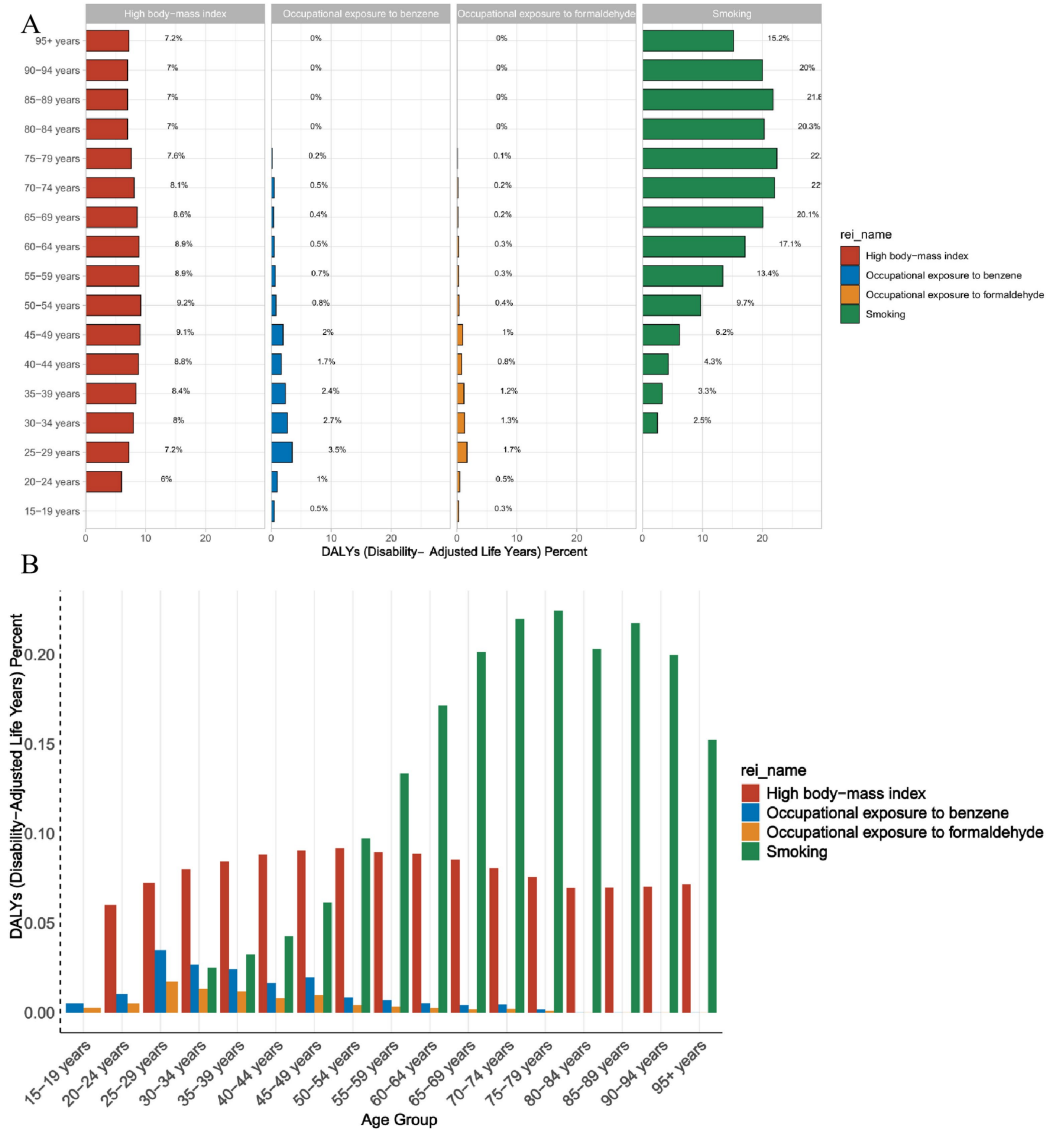
Age-specific distribution of leukemia DALYs attributable to selected risk factors in China, 2021: **(A)** percentages of leukemia DALYs attributable to high body-mass index, occupational exposure to benzene, occupational exposure to formaldehyde, and smoking across age groups; **(B)** comparative age-specific patterns of attributable DALY percentages for the four risk factors. Results are shown for both sexes combined. DALYs, disability-adjusted life years.

Table 3 details the contributions of DALYs attributable to smoking, high body-mass index, occupational exposure to benzene, and occupational exposure to formaldehyde for leukemia and its subtypes in China in 1990 and 2021. Among the selected risk factors, smoking was the largest contributor to leukemia-related DALYs. In 2021, smoking accounted for 187,807 attributable DALYs from leukemia (95% UI: 71,110, 329,568), with an age-standardized DALY rate of 8.68 per 100,000 population (95% UI: 3.28, 15.05), and the highest rate was observed in the 75–79 age group. High body-mass index ranked second, accounting for 144,005 attributable DALYs in 2021 (95% UI: 96,942, 198,783), with an age-standardized DALY rate of 7.82 per 100,000 population (95% UI: 5.25, 10.83), and the highest proportion was observed among those aged 50–54 years. In contrast, the attributable burdens from occupational exposure to benzene and formaldehyde were smaller, corresponding to age-standardized DALY rates of 1.45 (95% UI: 0.41, 2.56) and 0.71 (95% UI: 0.49, 0.93) per 100,000 population, respectively (Table 3 and Figure 8).

**Table 3:** DALYs attributable to four GBD-estimated risk factors for leukemia and its major subtypes in China, 1990 and 2021.

Cause	Risk factors	1990		2021		1990–2021		
		DALYs cases (95% UI)	ASR of DALYs (95% UI)	DALYs cases (95% UI)	ASR of DALYs (95% UI)	EAPCs (95% CI)	AAPC (95% CI)	*P*
Leukemia	High body-mass index	81045 (55536 to 113617)	7.38 (5.07 to 10.37)	144005 (96942 to 198783)	7.82 (5.25 to 10.83)	0.15 (0.09,0.22)	0.20 (0.02, 0.37)	0.028
Leukemia	Occupational exposure to benzene	18971 (5488 to 31673)	1.5 (0.44 to 2.51)	22460 (6316 to 39695)	1.45 (0.41 to 2.56)	−0.3 (−0.4,−0.19)	−0.09 (−0.38, 0.21)	0.570
Leukemia	Occupational exposure to formaldehyde	9258 (6708 to 11712)	0.73 (0.54 to 0.93)	10961 (7612 to 14359)	0.71 (0.49 to 0.93)	−0.26 (−0.39,−0.13)	−0.08 (−0.44, 0.28)	0.675
Leukemia	Smoking	91151 (37108 to 150052)	10.15 (4.12 to 16.73)	187807 (71110 to 329568)	8.68 (3.28 to 15.05)	−0.37 (−0.43,−0.3)	−0.52 (−0.65, −0.39)	<0.001
AML	High body-mass index	17203 (8926 to 28116)	1.6 (0.86 to 2.59)	36806 (23354 to 56606)	1.98 (1.25 to 3.01)	0.52 (0.34,0.7)	0.70 (0.49, 0.91)	<0.001
AML	Occupational exposure to benzene	3863 (1047 to 7348)	0.31 (0.08 to 0.59)	5700 (1577 to 10046)	0.37 (0.1 to 0.64)	0.31 (0.05,0.58)	0.57 (0.40, 0.74)	<0.001
AML	Occupational exposure to formaldehyde	1892 (928 to 2926)	0.15 (0.08 to 0.23)	2744 (1786 to 3907)	0.18 (0.11 to 0.25)	0.29 (−0.02,0.6)	0.53 (0.34, 0.72)	<0.001
AML	Smoking	20282 (6820 to 37075)	2.29 (0.79 to 4.19)	46222 (16687 to 85952)	2.14 (0.76 to 3.96)	−0.3 (−0.39,−0.21)	−0.24 (−0.43, −0.06)	0.010
CML	High body-mass index	5710 (2460 to 9437)	0.52 (0.22 to 0.85)	4630 (2580 to 8124)	0.25 (0.14 to 0.44)	−3.03 (−3.32,−2.74)	−2.32 (−2.72, −1.92)	<0.001
CML	Occupational exposure to benzene	1341 (377 to 2487)	0.11 (0.03 to 0.2)	716 (193 to 1377)	0.05 (0.01 to 0.09)	−3.73 (−4.11,−3.35)	−2.75 (−3.13, −2.36)	<0.001
CML	Occupational exposure to formaldehyde	663 (295 to 1048)	0.05 (0.02 to 0.08)	349 (202 to 590)	0.02 (0.01 to 0.04)	−3.74 (−4.13,−3.35)	−2.77 (−3.12, −2.42)	<0.001
CML	Smoking	5816 (1120 to 11267)	0.65 (0.13 to 1.24)	5781 (1411 to 13380)	0.27 (0.07 to 0.62)	−3.08 (−3.29,−2.88)	−2.83 (−3.18, −2.47)	<0.001
ALL	High body-mass index	30411 (19016 to 45059)	2.65 (1.67 to 3.91)	47370 (23988 to 68003)	2.68 (1.37 to 3.84)	0.09 (0.02,0.16)	0.05 (−0.06, 0.16)	0.367
ALL	Occupational exposure to benzene	8248 (2386 to 14226)	0.64 (0.18 to 1.1)	8492 (2213 to 15342)	0.57 (0.15 to 1.03)	−0.5 (−0.57,−0.42)	−0.33 (−0.53, −0.13)	<0.001
ALL	Occupational exposure to formaldehyde	4018 (2764 to 5524)	0.31 (0.21 to 0.43)	4162 (2340 to 5708)	0.28 (0.15 to 0.39)	−0.44 (−0.51,−0.37)	−0.29 (−0.47, −0.11)	0.002
ALL	Smoking	27514 (10650 to 48250)	2.95 (1.15 to 5.18)	54089 (17718 to 102791)	2.5 (0.82 to 4.75)	−0.17 (−0.32,−0.02)	−0.55 (−0.64, −0.46)	<0.001
CLL	High body-mass index	8959 (5049 to 13618)	0.87 (0.5 to 1.31)	22297 (13409 to 34248)	1.15 (0.69 to 1.77)	0.91 (0.84,0.99)	0.93 (0.81, 1.05)	<0.001
CLL	Occupational exposure to benzene	1676 (435 to 2934)	0.14 (0.04 to 0.24)	2795 (764 to 5103)	0.17 (0.05 to 0.3)	0.51 (0.39,0.63)	0.60 (0.31, 0.89)	<0.001
CLL	Occupational exposure to formaldehyde	813 (459 to 1190)	0.07 (0.04 to 0.1)	1380 (884 to 1961)	0.08 (0.05 to 0.12)	0.61 (0.46,0.75)	0.68 (0.38, 0.97)	<0.001
CLL	Smoking	13863 (4026 to 24762)	1.61 (0.47 to 2.87)	36920 (11130 to 75056)	1.7 (0.51 to 3.43)	0.26 (0.2,0.32)	0.17 (0.01, 0.33)	0.032

DALYs and age-standardized DALY rates are presented with 95% UIs. EAPCs and AAPCs are presented with 95% CIs. The *p*-value refers to the AAPC. DALYs, disability-adjusted life years; ASR, age-standardized rate; EAPC, estimated annual percentage change; AAPC, average annual percentage change; UI, uncertainty interval; CI, confidence interval.

Trend analysis (Table 3 and Supplementary Figure S5) showed that, from 1990 to 2021, CML exhibited the largest average annual decline in age-standardized DALY rates attributable to the four selected risk factors. In contrast, CLL showed increases in age-standardized DALY rates across all age groups, with the 75–79 age group contributing the most (Supplementary Figure S6). AML showed increases in age-standardized DALY rates attributable to high body-mass index, occupational exposure to benzene, and occupational exposure to formaldehyde, with AAPCs of 0.70, 0.57, and 0.53%, respectively; the largest increase attributable to high body-mass index occurred between 1997 and 2000. For ALL, the age-standardized DALY rate attributable to high body-mass index showed a slight increase. Sex-specific analyses (Supplementary Table S8) further indicated that, although the leukemia burden attributable to smoking among males showed a downward trend between 1990 and 2021, the age-standardized DALY rate remained relatively high.

## 4 Discussion

Based on the GBD 2021 estimates, our study highlights several distinctive features of leukemia epidemiology in China. Unlike global analyses that have consistently identified AML as the leading contributor to leukemia incidence and mortality (23), our findings demonstrate that ALL imposes the heaviest burden in China, particularly among children. This contrast underscores significant regional heterogeneity in leukemia subtypes, which is also supported by GLOBOCAN 2022 data showing pronounced variation in subtype distribution across countries (3). Such differences are likely shaped by a complex interplay of genetic susceptibility, environmental exposures, population aging, diagnostic capacity, and health system factors (24–26). Collectively, these results indicate that leukemia control in China requires approaches that go beyond a uniform framework and instead adopt differentiated, life-course strategies, prioritizing pediatric ALL management and targeted interventions for older adults with CLL.

Moreover, the higher burden in males compared with females observed in our study is consistent with global epidemiological patterns (27). Such sex disparities are increasingly recognized to reflect the interplay of multiple mechanisms. Among the various mechanisms, immune surveillance differences associated with sex chromosomes are likely to represent one of the key contributors. Females benefit from the presence of two X chromosomes, with approximately 15% of X-linked immune genes escaping inactivation, thereby strengthening anti-tumor immunity. In contrast, males not only lack this immunological advantage but also frequently exhibit Y-chromosome loss in hematopoietic and immune cells, which disrupts immune regulation and promotes leukemogenesis (28). Beyond immune regulation, related studies have shown sex-specific mutational patterns in acute myeloid leukemia and related subtypes. Mutations such as ASXL1, SRSF2, and RUNX1 are more common in males, whereas NPM1, FLT3, and TP53 mutations occur more frequently in females (29). These distinct genomic profiles suggest that genetic and epigenetic heterogeneity may further reinforce sex-related disparities in leukemogenesis and clinical outcomes. In addition, population-based evidence from China indicates that occupational exposure also contributes to this gap, as the Shanghai Men’s Health Study reported that high cumulative benzene exposure (>550 mg/m^3^) was significantly associated with increased leukemia risk among men (30). Collectively, these findings highlight the need to strengthen occupational health surveillance and implement stricter regulations on industrial carcinogen exposure in order to mitigate the sex-related disparities in leukemia burden.

Among the four major subtypes, CML showed the most substantial decline in incidence, mortality, and DALYs, a trend that is consistent with global estimates from GBD 2017 (5). This decline may be associated with advances in molecular diagnostics and the introduction of tyrosine kinase inhibitors (TKIs), which have dramatically improved long-term survival and transformed CML into a manageable chronic condition (31, 32). In China, the inclusion of TKIs in the national health insurance scheme further enhanced accessibility and equity, representing a major policy achievement. However, recent studies highlight persistent challenges, including poor health literacy, gaps in patient and caregiver knowledge, financial concerns, and psychological distress, all of which may compromise treatment adherence (33). These findings suggest that sustaining progress in CML control requires not only continued access to precision therapies but also comprehensive patient education, psychosocial support, and strategies to reduce socioeconomic barriers.

In contrast to the global trend of rising AML incidence (34), our analysis revealed a long-term decline in China. This divergence may be partly explained by stricter environmental regulations, reductions in occupational and chemical exposures, and improvements in cancer surveillance and registration systems (35, 36). For example, long-standing evidence links exposures such as formaldehyde and benzene to leukemogenic chromosomal alterations, and the gradual reduction of these occupational hazards provides a possible explanation for the observed decrease (37). Despite these encouraging declines, AML remained the second leading contributor to leukemia burden in China in 2021, underscoring the need for sustained prevention efforts. Strengthening occupational health protections and environmental exposure control remains essential, particularly given the continuing industrialization and urbanization pressures.

ALL exhibits a distinct incidence peak among children under 5 years of age, suggesting that the disease may have a specific biological basis related to early life. Multiple mechanisms may contribute to this age-specific pattern. First, genetic susceptibility plays a significant role in pediatric ALL. Genome-wide association studies (GWAS) have identified common allelic variants in genes such as IKZF1, ARID5B, CEBPE, and CDKN2A, which are involved in the regulation of hematopoiesis and lymphocyte development and are significantly associated with the risk of pediatric ALL. Multiple low-risk alleles can produce cumulative effects, thereby increasing individual susceptibility (38–40). Furthermore, genetic syndromes can also significantly increase the risk of pediatric ALL; for example, children with Down syndrome have a markedly higher risk of developing ALL than the general pediatric population (41). Second, pediatric ALL may originate from genetic events occurring in utero or during early life. Fusion gene formation, hyperdiploidy, or other initial alterations can generate occult preleukemic clones during the fetal period. These clones may remain dormant for extended periods; after birth, they may undergo secondary genetic events or be driven toward malignant transformation by dysregulated immune responses to common infections or chronic inflammation, ultimately progressing to leukemia (42). Recent studies suggest that factors such as cesarean delivery, reduced breastfeeding, and lack of social interaction may contribute to susceptibility to childhood ALL by influencing gut microbiota maturation, immune development, and patterns of infection exposure (43). Regarding environmental exposures, benzene and ionizing radiation are two environmental factors closely associated with the development of childhood ALL. Associations with other risk factors, such as pesticides and non-ionizing radiation, are weaker and inconsistent and require further validation (44).

The burden of CLL continues to increase among older adults, which may be related to age-associated alterations in the immune and hematopoietic systems, as well as the higher prevalence of comorbidities and reduced treatment tolerance in elderly patients. With advancing age, the immune system undergoes age-related remodeling, characterized mainly by impaired immune surveillance and chronic low-grade inflammation, which may provide a permissive background for the survival and expansion of abnormal B-cell clones (45, 46). Aging is also accompanied by accumulated somatic alterations and increased clonal hematopoiesis. Recent studies suggest that clonal hematopoiesis is relatively common in patients with CLL and may reflect genomic and inflammatory remodeling of the aging hematopoietic system (47). Furthermore, elderly patients with CLL often present with multiple comorbidities, frailty, impaired functional status, and reduced organ and bone marrow reserve; these factors may limit treatment tolerance, affect long-term outcomes, and further exacerbate the disease burden (48). However, analyses based on GBD data primarily reflect epidemiological trends in disease burden and cannot directly establish causal links between these biological or clinical mechanisms and the observed increase in CLL burden.

Joinpoint regression analysis indicated overall declining trends for AML and CML in China between 1990 and 2021, with AML showing a particularly steep reduction from 2004 to 2014 before stabilizing. This inflection in trend may be linked to advances in clinical management, including optimized chemotherapy regimens for younger patients, broader application of allogeneic hematopoietic stem cell transplantation (allo-HSCT), and the introduction of targeted therapies for elderly patients with elevated white blood cell counts, which together have improved survival outcomes (49). CML also demonstrated long-term declines, likely reflecting improvements in environmental exposures, early detection, and advances in treatment strategies. Importantly, the introduction of BCR-ABL1 TKIs in the early 2000s represented a landmark in the management of Philadelphia chromosome–positive CML, yielding substantial gains in survival and disease control (50). However, our analysis also revealed sex-specific inflections. AML incidence showed a minor increase among females after 2014, plausibly attributable to improved diagnostic sensitivity, whereas CML incidence began to rise among males after 2015, potentially linked to treatment adherence, occupational exposures, or other sex-specific risk factors. These findings suggest that while clinical innovations have driven overall declines in leukemia burden, aggregate trends may obscure subgroup-specific risks, highlighting the need for continuous sex-stratified monitoring.

The APC analysis provided further mechanistic insights into the heterogeneous trajectories of leukemia subtypes. As expected, the age effect was the dominant determinant across all subtypes. Risk rose steadily with advancing age, while ALL exhibited the highest relative risk in early childhood. Biological mechanisms may underlie these patterns. In children, immature immune systems and abnormal responses to early-life infections have been implicated in ALL susceptibility, along with recurrent germline alterations and gene rearrangements such as MLL translocation (51, 52). In elderly populations, clonal hematopoiesis driven by age-related somatic mutations, immune senescence, and progenitor cell remodeling contribute to increased risk of AML and CML (53–55). Period effects diverged by subtype: AML, CML, and ALL showed declining risks, consistent with in environmental exposures, and expanded healthcare coverage, whereas CLL exhibited a steady increase, likely reflecting enhanced diagnostic intensity and greater use of routine blood testing in the elderly (56, 57). Cohort effects revealed further contrasts. For AML, CML, and CLL, individuals born in earlier cohorts showed higher risks compared to those born in more recent cohorts. One well-characterized example is ionizing radiation, an established leukemogenic exposure. In the early 20th century, limited understanding of radiation hazards led to widespread and often unprotected exposure to X-rays, radium, and other radioactive materials. Historical evidence, including increased leukemia incidence among atomic bomb survivors in Hiroshima and Nagasaki, provides compelling support for radiation as a key early-life leukemogenic exposure (58). By contrast, ALL demonstrated a modest rise among cohorts born after 2007. Although evidence from China is still scarce, studies in other regions have shown that adolescents often have insufficient environmental health literacy, with limited awareness of leukemia-related hazards such as benzene, pesticides, and electromagnetic radiation (59). Together, these findings indicate that while age remains the strongest determinant of leukemia risk, period- and cohort-specific dynamics uniquely shape each subtype, highlighting the need for strategies that simultaneously address generational exposures and system-level improvements.

Compared with previous BAPC-based projections of leukemia subtypes, CLL showed the most pronounced divergence. Although some global forecasting studies have projected a gradual decline in the overall ASIR of hematologic malignancies, CLL-specific BAPC studies have reported that ASIR may continue to increase in several countries, including China, India, Afghanistan, and Ukraine (60, 61). Our findings are broadly consistent with these country-level projections, suggesting that CLL may follow a more region-specific trajectory than other leukemia subtypes. Notably, both the ASIR and the absolute number of incident CLL cases in China are projected to increase. These projections underscore the urgent need to strengthen preventive and control strategies for older adults, particularly those aged 75 years and above, who represent the most vulnerable population segment. Key actions include enhancing early detection through routine blood tests and molecular diagnostic tools, expanding public health awareness, and ensuring equitable access to advanced diagnostics and treatment in underserved areas (49). Furthermore, unlike the declining trend in AML ASIR reported in previous global studies, our study projects only a slight increase in AML ASIR in China, while the absolute number of incident cases is expected to continue rising (62). The projected ASIR trends for CML and ALL are generally consistent with previous global downward trends (60, 63). However, the absolute number of incident CML cases may still increase further. Continuous epidemiological surveillance is therefore essential to monitor evolving patterns and inform evidence-based policies.

In summary, this study demonstrates that the epidemiological profile of leukemia subtypes in China diverges substantially from global patterns. While AML remains the leading contributor worldwide, China exhibits a bimodal pattern of burden, characterized by the predominance of ALL in children and a steady increase of CLL among older adults. The marked decline in CML may reflect the broader availability of precision therapies and policy interventions, particularly the incorporation of TKIs into the national health insurance scheme (64), whereas the reduction in AML burden reflects improvements in occupational and environmental control. These findings suggest that effective leukemia control in China requires a dual strategy: sustaining progress in pediatric ALL management and incorporating CLL into chronic disease programs targeting the elderly. At the national level, these implications align with the Healthy China 2030 agenda, the 2019 National Action for Pediatric Leukemia and Childhood Tumor Treatment, and the 14th Five-Year Plan for Healthy Aging. At the global level, the high burden of childhood ALL is consistent with the World Health Organization (WHO) Global Initiative for Childhood Cancer, while the overall decline in leukemia mortality contributes to the achievement of Sustainable Development Goal 3.4, which calls for a one-third reduction in premature mortality from non-communicable diseases by 2030. These policy linkages underscore the broader clinical and public health relevance of our findings and the necessity of adopting differentiated, life-course strategies for leukemia prevention and control.

This study has several limitations. First, the data sources and the model estimates may lead to bias. This study was based on estimates from GBD 2021 rather than original cancer registry data or individual patient-level data. Previous methodological studies have noted that uncertainty in burden estimates may arise not only from the input data themselves but also from the modeling procedures and assumptions. Such uncertainty may be further amplified when available data are limited, when discrepancies exist across data sources, or when sample sizes are small (65). In addition, GBD estimates often require extrapolation from limited data sources to larger populations, which may introduce selection bias, measurement error, and systematic error (66). Consequently, for certain leukemia subtypes—particularly in earlier years, in groups with sparse data, or during periods when diagnostic and classification criteria changed—the disease burden may have been overestimated or underestimated to some extent. Second, GBD 2021 lacked subnational data on the disease burden of leukemia subtypes in China, making provincial comparisons impossible. This limitation is worth noting because there may be significant differences across China’s provinces and between urban and rural areas in terms of social and economic development, the quality of cancer registries, diagnostic capabilities, and access to treatment. Consequently, the national aggregate results may mask domestic variation and limit the practical guidance value for provincial health management. Third, the lack of immunophenotypic, cytogenetic, molecular genetic, and patient-level clinical information in the GBD database limited the clinical interpretation of our findings. Leukemia subtypes are highly heterogeneous, and their diagnostic classification, risk stratification, treatment selection, and prognostic assessment rely on bone marrow morphology, immunophenotyping, karyotyping/fluorescence *in situ* hybridization (FISH), fusion gene and mutation testing, as well as clinical information on treatment regimens, treatment response, relapse, and survival outcomes. However, these granular variables are not available in the GBD framework. Therefore, although this study described population-level trends in the burden of leukemia subtypes, it could not determine the extent to which these trends were influenced by genetic risk profiles, disease stratification, treatment accessibility, or differences in patient prognosis. Nor can these findings be directly used to explain individual clinical outcomes or specific biological mechanisms. Fourth, the BAPC projections should be understood as conditional estimates based on the assumption that past trends will continue, rather than precise predictions of the future disease burden. Although the model can reflect a certain degree of statistical uncertainty through its prediction intervals, it is difficult to fully capture the structural uncertainty arising from external factors such as the future adoption of novel targeted therapies, adjustments to health insurance policies, improvements in treatment accessibility, and changes in demographic structure. In addition, because this study was based on observational, modeled estimates from the GBD database, it can only describe associations between changes in disease burden and potential contributing factors, rather than establish strict causal relationships. Therefore, the interpretations regarding possible drivers of leukemia subtype trends should be considered hypothesis-generating. Future real-world studies, registry-based analyses, and patient-level cohort studies are needed to further validate these findings and clarify the underlying mechanisms.

## 5 Conclusion

In China, leukemia subtypes exhibit distinct long-term trends in incidence and disease burden. While the burdens of AML and CML have declined steadily, the increasing incidence of ALL and CLL poses emerging challenges, especially in children and the elderly populations. Age remains the dominant risk factor, and anticipated increases in CLL underscore the need for proactive planning in geriatric cancer care. These findings highlight the importance of subtype-specific, age-tailored, and forward-looking strategies to reduce the future burden of leukemia in China.

## Data Availability

The datasets presented in this study can be found in online repositories. The names of the repository/repositories and accession number(s) can be found in the article/Supplementary material.

## Footnotes

1
http://ghdx.healthdata.org/gbd-results-tool

## Supplementary material

SUPPLEMENTARY FIGURE S1 Trends in the age-standardized rates of incidence for leukemia subtypes in China by sex, 1990–2021. **(A)** AML; **(B)** CML; **(C)** ALL; **(D)** CLL. Lines represent ASRs for both sexes (red), females (green), and males (blue). Shaded areas indicate the 95% uncertainty intervals (UIs). ASR, age-standardized rate per 100,000 population. Data were obtained from the Global Burden of Disease Study 2021.

SUPPLEMENTARY FIGURE S2 Trends in the age-standardized rates of deaths for leukemia subtypes in China by sex, 1990–2021. **(A)** AML; **(B)** CML; **(C)** ALL; **(D)** CLL. Lines represent ASRs for both sexes (red), females (green), and males (blue). Shaded areas indicate the 95% uncertainty intervals (UIs). ASR, age-standardized rate per 100,000 population. Data were obtained from the Global Burden of Disease Study 2021.

SUPPLEMENTARY FIGURE S3 Trends in the age-standardized rates of DALYs for leukemia subtypes in China by sex, 1990–2021. **(A)** AML; **(B)** CML; **(C)** ALL; **(D)** CLL. Lines represent ASRs for both sexes (red), females (green), and males (blue). Shaded areas indicate the 95% uncertainty intervals (UIs). DALYs, disability-adjusted life-years; ASR, age-standardized rate per 100,000 population. Data were obtained from the Global Burden of Disease Study 2021.

SUPPLEMENTARY FIGURE S4 Predicted incidence case numbers for leukemia subtypes in China from 1990 to 2046. **(A)** AML; **(B)** CML; **(C)** ALL; **(D)** CLL. Results are shown for both sexes combined. The solid lines represent observed values from 1990 to 2021, while the dashed lines indicate projections from 2022 to 2046 based on the Bayesian age–period–cohort model. Shaded areas denote the 95% uncertainty intervals (UIs).

SUPPLEMENTARY FIGURE S5 Joinpoint regression analysis of age-standardized DALY rates attributable to four risk factors for leukemia subtypes in China, 1990–2021. Trends are shown for **(A)** AML, **(B)** CML, **(C)** ALL, and **(D)** CLL for both sexes combined. Lines indicate segmented trends estimated by Joinpoint regression. APC denotes annual percent change, and * indicates statistical significance at *p* < 0.05. APC, annual percent change; DALYs, disability-adjusted life years; AML, acute myeloid leukemia; CML, chronic myeloid leukemia; ALL, acute lymphoblastic leukemia; CLL, chronic lymphocytic leukemia.

SUPPLEMENTARY FIGURE S6 Age-specific proportions of DALYs attributable to four GBD-estimated risk factors for leukemia subtypes in China, 2021. Age-specific percentages of attributable DALYs are shown for **(A)** AML, **(B)** CML, **(C)** ALL, and **(D)** CLL among both sexes combined. Bars represent point estimates of attributable DALY percentages. DALYs, disability-adjusted life years; AML, acute myeloid leukemia; CML, chronic myeloid leukemia; ALL, acute lymphoblastic leukemia; CLL, chronic lymphocytic leukemia.
