# Supplementary figures and images for: Long-term trends in the burden of leukemia subtypes in China from 1990 to 2021: a Joinpoint regression and age-period-cohort analysis based on GBD 2021
Source: Front Med (Lausanne). 2026 Jun 4;13:1826237. doi: 10.3389/fmed.2026.1826237 (PMC13275245; doi:10.3389/fmed.2026.1826237)

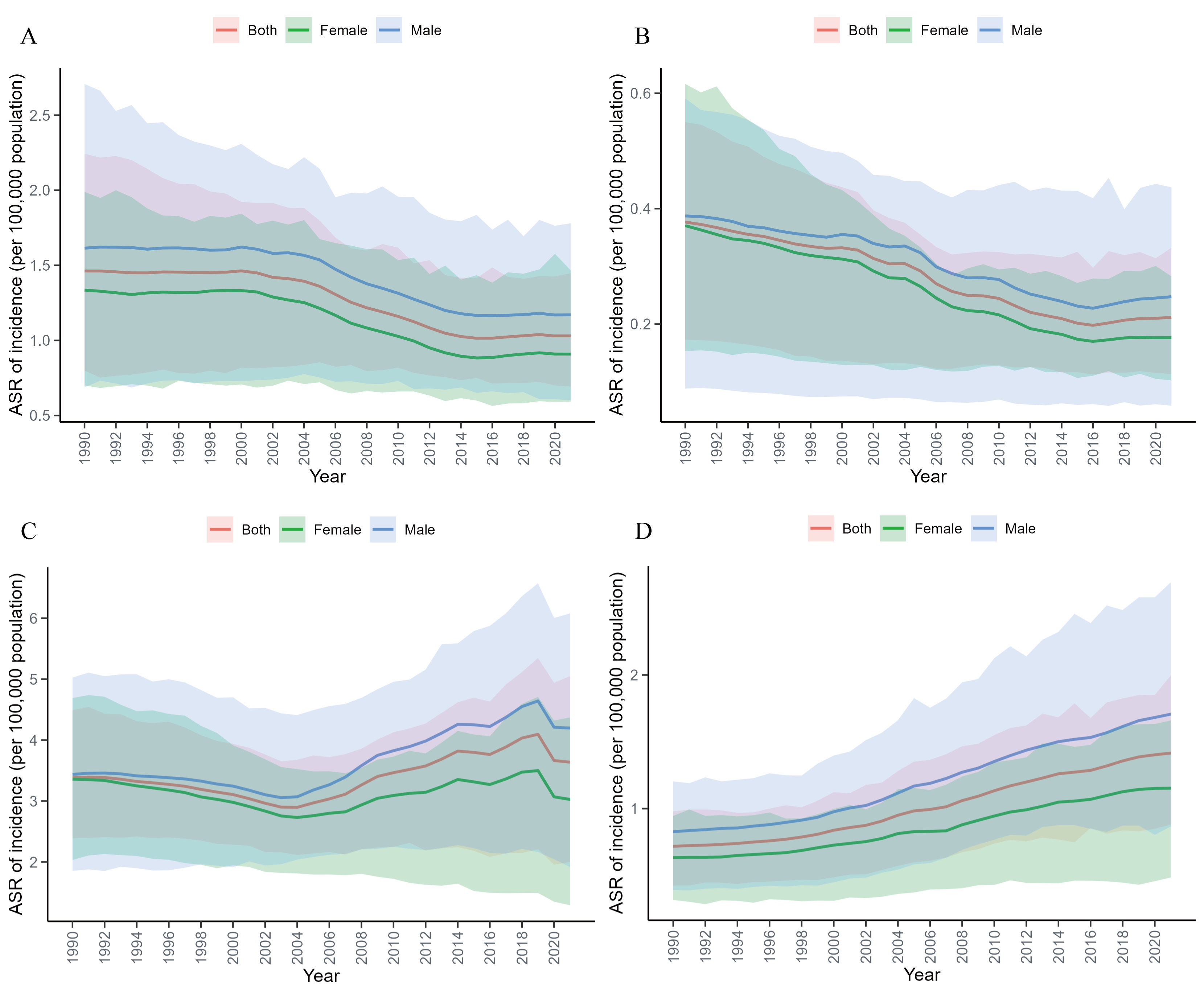

Supplement: SUPPLEMENTARY FIGURE S1 — Trends in the age-standardized rates of incidence for leukemia subtypes in China by sex, 1990–2021. (A) AML; (B) CML; (C) ALL; (D) CLL. Lines represent ASRs for both sexes (red), females (green), and males (blue). Shaded areas indicate the 95% uncertainty intervals (UIs). ASR, age-standardized rate per 100,000 population. Data were obtained from the Global Burden of Disease Study 2021. [file Image_1.tif]

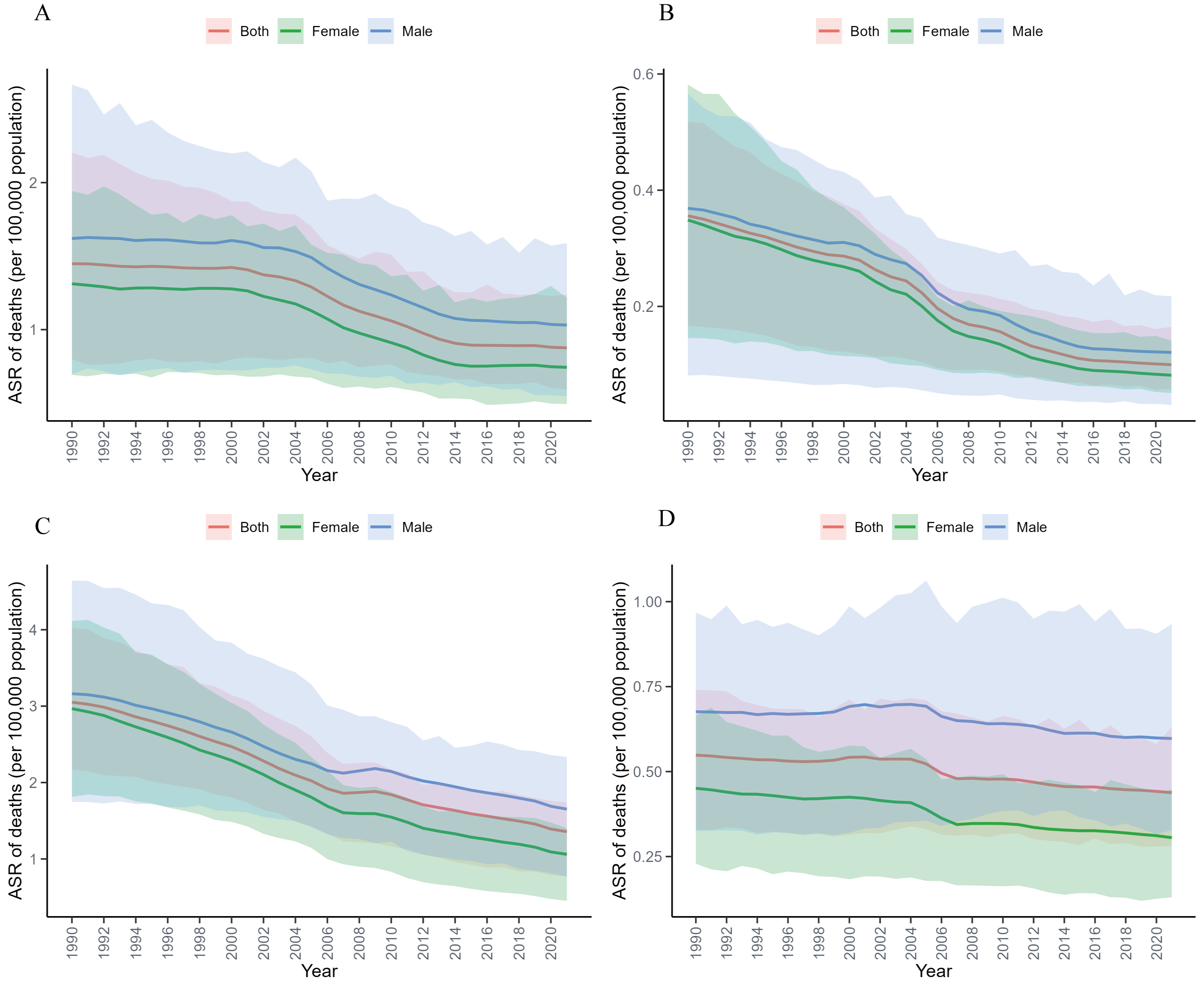

Supplement: SUPPLEMENTARY FIGURE S2 — Trends in the age-standardized rates of deaths for leukemia subtypes in China by sex, 1990–2021. (A) AML; (B) CML; (C) ALL; (D) CLL. Lines represent ASRs for both sexes (red), females (green), and males (blue). Shaded areas indicate the 95% uncertainty intervals (UIs). ASR, age-standardized rate per 100,000 population. Data were obtained from the Global Burden of Disease Study 2021. [file Image_2.tif]

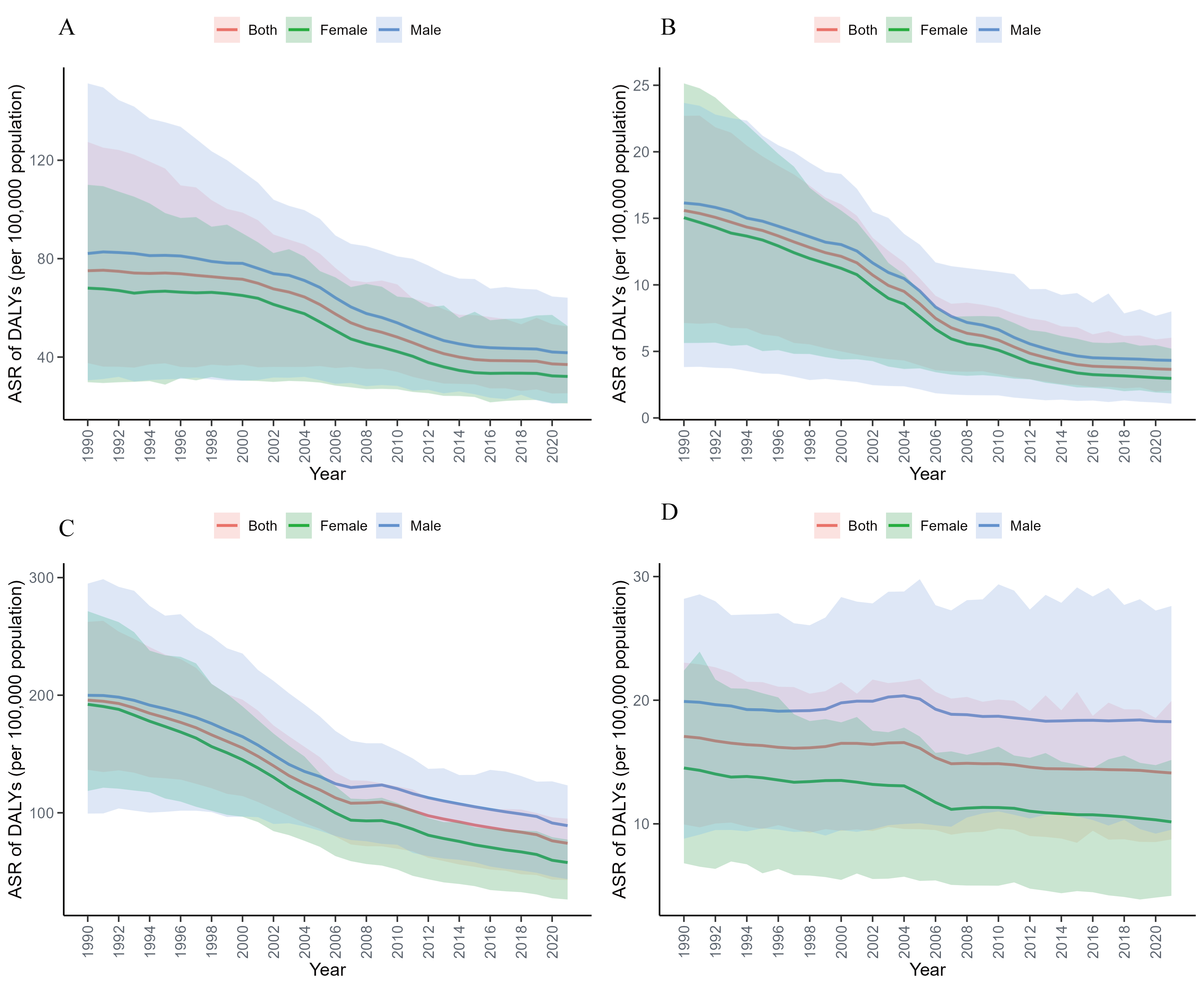

Supplement: SUPPLEMENTARY FIGURE S3 — Trends in the age-standardized rates of DALYs for leukemia subtypes in China by sex, 1990–2021. (A) AML; (B) CML; (C) ALL; (D) CLL. Lines represent ASRs for both sexes (red), females (green), and males (blue). Shaded areas indicate the 95% uncertainty intervals (UIs). DALYs, disability-adjusted life-years; ASR, age-standardized rate per 100,000 population. Data were obtained from the Global Burden of Disease Study 2021. [file Image_3.tif]

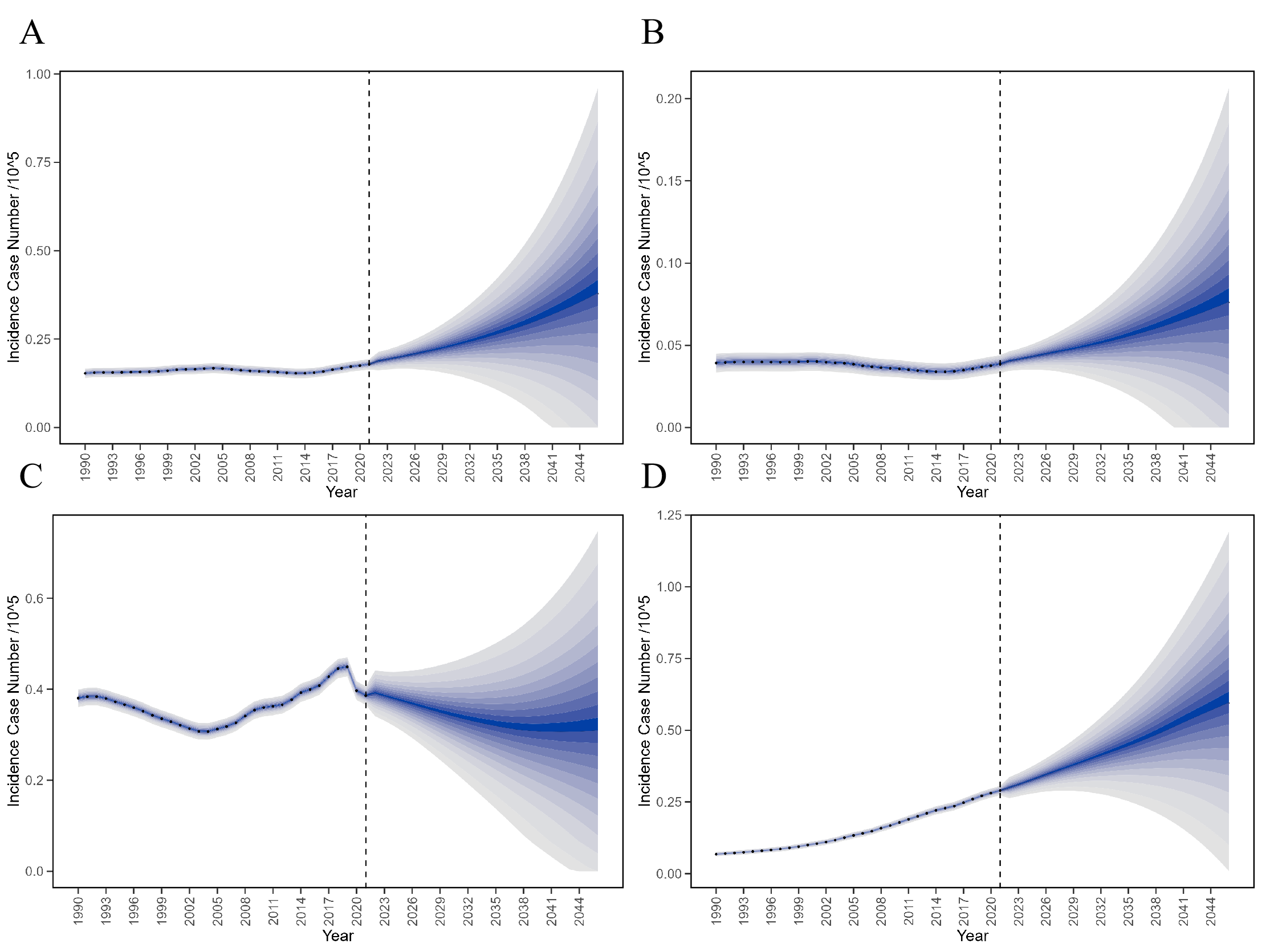

Supplement: SUPPLEMENTARY FIGURE S4 — Predicted incidence case numbers for leukemia subtypes in China from 1990 to 2046. (A) AML; (B) CML; (C) ALL; (D) CLL. Results are shown for both sexes combined. The solid lines represent observed values from 1990 to 2021, while the dashed lines indicate projections from 2022 to 2046 based on the Bayesian age–period–cohort model. Shaded areas denote the 95% uncertainty intervals (UIs). [file Image_4.tif]

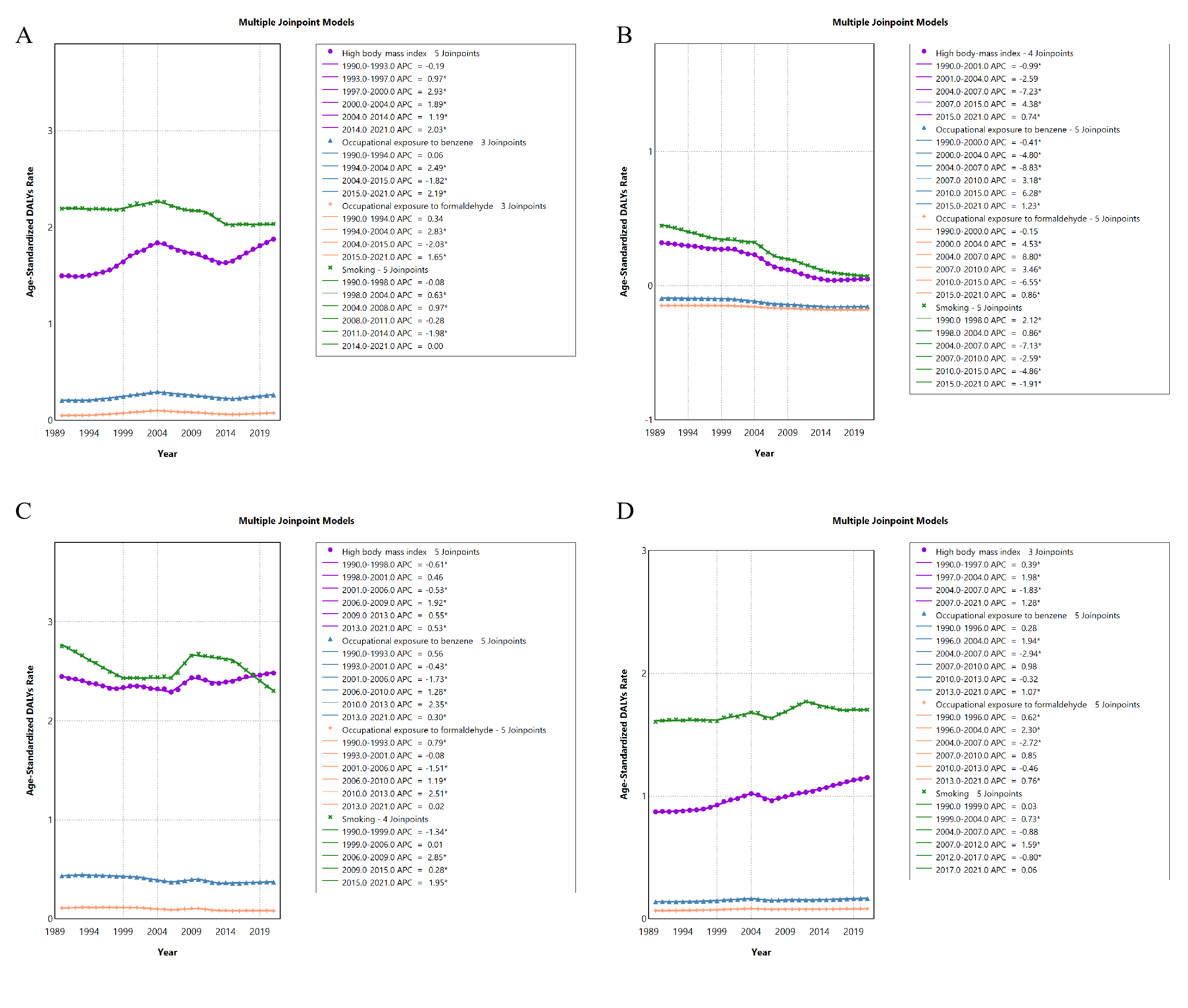

Supplement: SUPPLEMENTARY FIGURE S5 — Joinpoint regression analysis of age-standardized DALY rates attributable to four risk factors for leukemia subtypes in China, 1990–2021. Trends are shown for (A) AML, (B) CML, (C) ALL, and (D) CLL for both sexes combined. Lines indicate segmented trends estimated by Joinpoint regression. APC denotes annual percent change, and * indicates statistical significance at p < 0.05. APC, annual percent change; DALYs, disability-adjusted life years; AML, acute myeloid leukemia; CML, chronic myeloid leukemia; ALL, acute lymphoblastic leukemia; CLL, chronic lymphocytic leukemia. [file Image_5.tif]

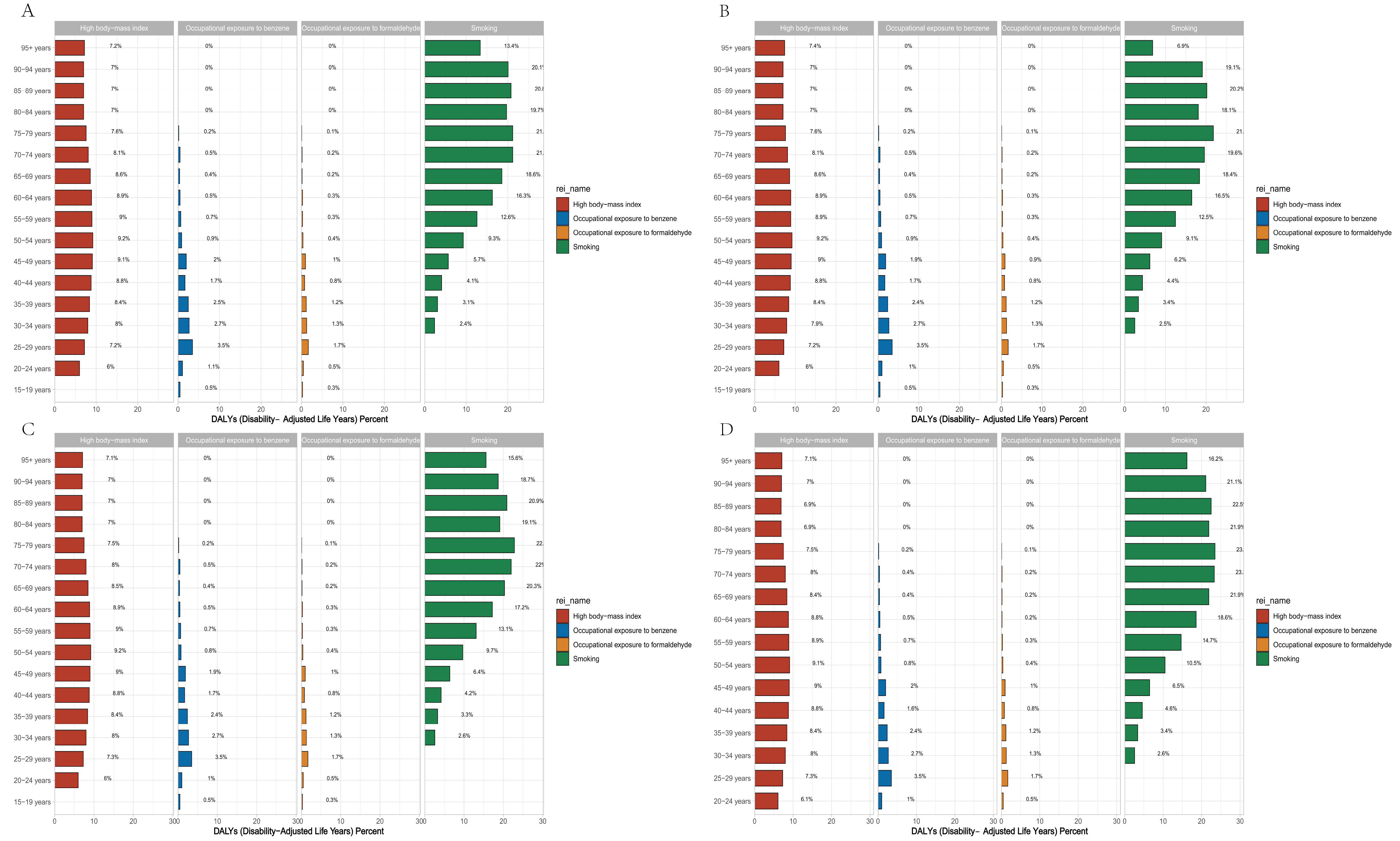

Supplement: SUPPLEMENTARY FIGURE S6 — Age-specific proportions of DALYs attributable to four GBD-estimated risk factors for leukemia subtypes in China, 2021. Age-specific percentages of attributable DALYs are shown for (A) AML, (B) CML, (C) ALL, and (D) CLL among both sexes combined. Bars represent point estimates of attributable DALY percentages. DALYs, disability-adjusted life years; AML, acute myeloid leukemia; CML, chronic myeloid leukemia; ALL, acute lymphoblastic leukemia; CLL, chronic lymphocytic leukemia. [file Image_6.tif]
