# Supplementary material for: Long-term trends in the burden of leukemia subtypes in China from 1990 to 2021: a Joinpoint regression and age-period-cohort analysis based on GBD 2021
Source: Front Med (Lausanne). 2026 Jun 4;13:1826237. doi: 10.3389/fmed.2026.1826237 (PMC13275245; doi:10.3389/fmed.2026.1826237)
Supplement: Supplementary file 7 [file Table_1.docx]

**Table S1.** The number and rate of incidence, deaths, and DALYs of leukemia and its subtypes by age group for both sexes in China, 2021.

| **Cause** | **Measure** | **<5 years** | | **5-14 years** | | **15-49 years** | | **50-69 years** | | **70+ years** | |
| --- | --- | --- | --- | --- | --- | --- | --- | --- | --- | --- | --- |
|  |  | **Number** | **Rate** | **Number** | **Rate** | **Number** | **Rate** | **Number** | **Rate** | **Number** | **Rate** |
| Leukemia | DALYs | 122777.88 (72577.31,183244.05) | 158.08 (93.45,235.93) | 870518.84 (632670.97,1089099.38) | 131.24 (95.38,164.19) | 240330.20 (170622.99,305661.49) | 132.08 (93.77,167.98) | 682862.43 (506762.44,887969.49) | 179.46 (133.18,233.36) | 288731.22 (216136.73,363978.23) | 242.01 (181.16,305.08) |
|  | Deaths | 1300.02 (777.85,1939.7) | 1.67 (1,2.5) | 15483.42 (11295.37,19418.26) | 2.33 (1.7,2.93) | 2941.08 (2091.77,3723.55) | 1.62 (1.15,2.05) | 21693.08 (15886.03,28132.22) | 5.70 (4.17,7.39) | 17485.87 (13241.01,21979.05) | 14.66 (11.1,18.42) |
|  | Incidence | 12668.65 (6522.06,20055.44) | 16.31 (8.4,25.82) | 27613.32 (19729.74,34642.05) | 4.16 (2.97,5.22) | 7652.54 (5046.86,10023.89) | 4.21 (2.77,5.51) | 37167.88 (26962.38,49446.68) | 9.77 (7.09,12.99) | 20564.80 (15577.37,25946.84) | 17.24 (13.06,21.75) |
| AML | DALYs | 29383.11 (16814.04,49008.3) | 37.83 (21.65,63.1) | 51502.31 (34521.53,84612.63) | 28.30 (18.97,46.5) | 214586.91 (141425.56,292582.2) | 32.35 (21.32,44.11) | 178108.29 (119342.45,243609.66) | 46.81 (31.36,64.02) | 74974.77 (51954.2,109228.72) | 62.84 (43.55,91.55) |
|  | Deaths | 331.58 (189.56,552.81) | 0.43 (0.24,0.71) | 639.46 (429.16,1047.85) | 0.35 (0.24,0.58) | 3867.86 (2552.91,5303.01) | 0.58 (0.38,0.8) | 5800.50 (3870.99,7919.06) | 1.52 (1.02,2.08) | 4671.71 (3245.01,6760.45) | 3.92 (2.72,5.67) |
|  | Incidence | 408.21 (234.2,675.16) | 0.53 (0.3,0.87) | 784.94 (520.26,1272.52) | 0.43 (0.29,0.7) | 5098.11 (3301.96,6962.53) | 0.77 (0.5,1.05) | 6857.49 (4534.69,9420.3) | 1.80 (1.19,2.48) | 4686.46 (3205.85,6676.41) | 3.93 (2.69,5.6) |
| CML | DALYs | 496.40 (190.71,1114.9) | 0.64 (0.25,1.44) | 2655.27 (1468.47,4985.05) | 1.46 (0.81,2.74) | 26790.63 (14428.53,43135.33) | 4.04 (2.18,6.5) | 20665.93 (11923.6,35437.57) | 5.43 (3.13,9.31) | 10320.43 (6274.29,17917.4) | 8.65 (5.26,15.02) |
|  | Deaths | 5.61 (2.15,12.62) | 0.01 (0,0.02) | 32.91 (18.22,61.77) | 0.02 (0.01,0.03) | 489.29 (263.93,795.71) | 0.07 (0.04,0.12) | 659.83 (379.4,1142.1) | 0.17 (0.1,0.3) | 653.04 (396.88,1146.74) | 0.55 (0.33,0.96) |
|  | Incidence | 13.92 (4.84,29.07) | 0.02 (0.01,0.04) | 81.42 (42.83,142.83) | 0.04 (0.02,0.08) | 1378.39 (676.74,2118.76) | 0.21 (0.1,0.32) | 1504.81 (810.94,2410.54) | 0.40 (0.21,0.63) | 871.32 (506.38,1447.68) | 0.73 (0.42,1.21) |
| ALL | DALYs | 89855.83 (46552.02,139487.33) | 115.69 (59.94,179.59) | 181873.20 (111614.58,236188.64) | 99.95 (61.34,129.8) | 376267.47 (206653.3,485047.86) | 56.73 (31.16,73.13) | 204695.00 (108747.27,279228.24) | 53.79 (28.58,73.38) | 71730.69 (38401,99903.72) | 60.12 (32.19,83.74) |
|  | Deaths | 930.64 (487.57,1433.88) | 1.20 (0.63,1.85) | 2217.79 (1360.02,2869.02) | 1.22 (0.75,1.58) | 6507.41 (3590.43,8422.55) | 0.98 (0.54,1.27) | 6578.13 (3479.22,9000.85) | 1.73 (0.91,2.37) | 4378.94 (2379.76,6066.36) | 3.67 (1.99,5.08) |
|  | Incidence | 11915.36 (5844.63,18968.8) | 15.34 (7.53,24.42) | 6462.22 (3835.86,8525.41) | 3.55 (2.11,4.69) | 9656.91 (5301.39,12582.4) | 1.46 (0.8,1.9) | 6953.39 (3524.2,9475.73) | 1.83 (0.93,2.49) | 3583.06 (1879.98,4986.72) | 3.00 (1.58,4.18) |
| CLL | DALYs | 0.00 (0,0) | 0.00 (0,0) | 0.00 (0,0) | 0.00 (0,0) | 89945.38 (54983.95,127379.93) | 13.56 (8.29,19.2) | 117965.11 (72284.96,166414.32) | 31.00 (19,43.73) | 60340.04 (39969.23,88141.3) | 50.58 (33.5,73.88) |
|  | Deaths | NA | NA | NA | NA | 1627.57 (998.68,2292.6) | 0.25 (0.15,0.35) | 3511.42 (2163.76,4979.83) | 0.92 (0.57,1.31) | 3497.08 (2388.16,5116.84) | 2.93 (2,4.29) |
|  | Incidence | 0.00 (0,0) | 0.00 (0,0) | 0.00 (0,0) | 0.00 (0,0) | 6518.63 (3946.97,9067.72) | 0.98 (0.6,1.37) | 14930.42 (9038.57,20911.32) | 3.92 (2.38,5.5) | 7477.66 (4839.46,10782.73) | 6.27 (4.06,9.04) |

Data are shown as numbers and rates per 100,000 population with 95% uncertainty intervals (UIs) in parentheses. DALYs, disability-adjusted life years; AML, acute myeloid leukemia; CML, chronic myeloid leukemia; ALL, acute lymphoblastic leukemia; CLL, chronic lymphocytic leukemia. Data source: Global Burden of Disease Study 2021.
