# Supplementary material for: Long-term trends in the burden of leukemia subtypes in China from 1990 to 2021: a Joinpoint regression and age-period-cohort analysis based on GBD 2021
Source: Front Med (Lausanne). 2026 Jun 4;13:1826237. doi: 10.3389/fmed.2026.1826237 (PMC13275245; doi:10.3389/fmed.2026.1826237)
Supplement: Supplementary file 8 [file Table_2.docx]

**Table S2.** Joinpoint regression analysis of age-standardized incidence rate (ASIR) of chronic myeloid leukemia (CML) in China, 1990–2021

| **sex** | **Segment(year)** | **APC(%)** | **95%CI** | **P-Value** |
| --- | --- | --- | --- | --- |
| **Both** | 1990-2004 | -1.49 | -1.62~-1.34 | <0001 |
|  | 2004-2007 | -5.85 | -6.46~-4.37 | <0001 |
|  | 2007-2010 | -1.8 | -3.01~-1.06 | <0001 |
|  | 2010-2015 | -3.86 | -4.88~-3.26 | 0.01 |
|  | 2015-2021 | 1.08 | 0.63~1.54 | <0001 |
|  | AAPC(%) | -1.845 | -1.91~-1.78 | <0001 |
| **Female** | 1990-2001 | -1.69 | -1.95~-1.38 | <0001 |
|  | 2001-2015 | -4 | -4.24~-3.81 | <0001 |
|  | 2015-2021 | 0.48 | -0.22~1.4 | 0.12 |
|  | AAPC(%) | -2.331 | -2.42~-2.25 | <0001 |
| **Male** | 1990-2004 | -1.04 | -1.23~-0.85 | 0.02 |
|  | 2004-2007 | -5.07 | -5.78~-1.05 | 0.01 |
|  | 2007-2010 | -1.4 | -3.83~-0.44 | 0.01 |
|  | 2010-2015 | -3.64 | -5.02~-2.66 | 0.03 |
|  | 2015-2021 | 1.41 | 0.85~2.33 | 0.01 |
|  | AAPC(%) | -1.429 | -1.51~-1.36 | <0001 |

APC, annual percentage change; AAPC, average annual percentage change; CI, confidence interval. Data are shown with 95% confidence intervals. Data source: Global Burden of Disease Study 2021.
