# Supplementary material for: Long-term trends in the burden of leukemia subtypes in China from 1990 to 2021: a Joinpoint regression and age-period-cohort analysis based on GBD 2021
Source: Front Med (Lausanne). 2026 Jun 4;13:1826237. doi: 10.3389/fmed.2026.1826237 (PMC13275245; doi:10.3389/fmed.2026.1826237)
Supplement: Supplementary file 9 [file Table_3.docx]

Table S3. Joinpoint regression analysis of age-standardized incidence rate (ASIR) of acute myeloid leukemia (AML) in China, 1990–2021

| **sex** | **Segment(year)** | **APC(%)** | **95%CI** | **P-Value** |
| --- | --- | --- | --- | --- |
| **Both** | 1990-2000 | 0 | -0.12~0.16 | 0.97 |
|  | 2000-2004 | -1.12 | -1.86~-0.65 | <0001 |
|  | 2004-2014 | -3.08 | -3.2~-2.97 | <0001 |
|  | 2014-2021 | 0.23 | 0.06~0.38 | 0.02 |
|  | AAPC(%) | -1.099 | -1.13~-1.06 | <0001 |
| **Female** | 1990-1993 | -0.68 | -1.53~0.03 | 0.06 |
|  | 1993-2000 | 0.29 | -1.47~0.82 | 0.07 |
|  | 2000-2004 | -1.66 | -3.3~-0.99 | <0001 |
|  | 2004-2014 | -3.35 | -3.47~-3.17 | 0.03 |
|  | 2014-2021 | 0.41 | 0.23~0.58 | 0.01 |
|  | AAPC(%) | -1.215 | -1.26~-1.17 | <0001 |
| **Male** | 1990-2001 | -0.08 | -0.17~0.07 | 0.23 |
|  | 2001-2004 | -0.89 | -2.79~-0.37 | <0001 |
|  | 2004-2014 | -2.88 | -3.01~-2.71 | 0.02 |
|  | 2014-2021 | 0.07 | -0.07~0.23 | 0.28 |
|  | AAPC(%) | -1.035 | -1.06~-1 | <0001 |

APC, annual percentage change; AAPC, average annual percentage change; CI, confidence interval. Data are shown with 95% confidence intervals. Data source: Global Burden of Disease Study 2021
