# Supplementary material for: Long-term trends in the burden of leukemia subtypes in China from 1990 to 2021: a Joinpoint regression and age-period-cohort analysis based on GBD 2021
Source: Front Med (Lausanne). 2026 Jun 4;13:1826237. doi: 10.3389/fmed.2026.1826237 (PMC13275245; doi:10.3389/fmed.2026.1826237)
Supplement: Supplementary file 10 [file Table_4.docx]

**Table S4.** Joinpoint regression analysis of age-standardized incidence rate (ASIR) of chronic lymphocytic leukemia(CLL) in China, 1990–2021

| **sex** | **Segment(year)** | **APC(%)** | **95%CI** | **P-Value** |
| --- | --- | --- | --- | --- |
| **Both** | 1990-1997 | 0.94 | 0.56~1.22 | <0001 |
|  | 1997-2013 | 3.04 | 2.95~3.14 | <0001 |
|  | 2013-2021 | 1.83 | 1.56~2.05 | <0001 |
|  | AAPC(%) | 2.249 | 2.2~2.3 | <0001 |
| **Female** | 1990-1997 | 0.87 | 0.5~1.15 | 0.01 |
|  | 1997-2004 | 2.72 | 1.34~3.36 | <0001 |
|  | 2004-2007 | 1.51 | 1.03~3.05 | <0001 |
|  | 2007-2011 | 3.8 | 1.57~4.48 | <0001 |
|  | 2011-2019 | 1.98 | 1.82~3.56 | <0001 |
|  | 2019-2021 | 0.42 | -0.44~1.71 | 0.28 |
|  | AAPC(%) | 1.981 | 1.92~2.04 | <0001 |
| **Male** | 1990-1998 | 1.22 | 0.89~1.47 | <0001 |
|  | 1998-2011 | 3.43 | 3.3~3.59 | <0001 |
|  | 2011-2021 | 2 | 1.79~2.17 | <0001 |
|  | AAPC(%) | 2.392 | 2.34~2.44 | <0001 |

APC, annual percentage change; AAPC, average annual percentage change; CI, confidence interval. Data are shown with 95% confidence intervals. Data source: Global Burden of Disease Study 2021
