# Supplementary material for: Long-term trends in the burden of leukemia subtypes in China from 1990 to 2021: a Joinpoint regression and age-period-cohort analysis based on GBD 2021
Source: Front Med (Lausanne). 2026 Jun 4;13:1826237. doi: 10.3389/fmed.2026.1826237 (PMC13275245; doi:10.3389/fmed.2026.1826237)
Supplement: Supplementary file 11 [file Table_5.docx]

**Table S5.** Joinpoint regression analysis of age-standardized incidence rate (ASIR) of acute lymphoblastic leukemia(ALL) in China, 1990–2021

| **sex** | **Segment(year)** | **APC(%)** | **95%CI** | **P-Value** |
| --- | --- | --- | --- | --- |
| **Both** | 1990-1997 | -0.65 | -1.05~0.31 | 0.09 |
|  | 1997-2004 | -1.81 | -3.19~-1.4 | 0.03 |
|  | 2004-2010 | 3.28 | 2.7~4.7 | <0001 |
|  | 2010-2019 | 1.69 | 1.31~2.01 | <0001 |
|  | 2019-2021 | -6.05 | -7.77~-3.34 | <0001 |
|  | AAPC(%) | 0.149 | 0.03~0.26 | 0.01 |
| **Female** | 1990-1997 | -1 | -1.59~0.2 | 0.07 |
|  | 1997-2004 | -2.13 | -3.6~0.42 | 0.07 |
|  | 2004-2014 | 1.99 | -1.65~3.33 | 0.08 |
|  | 2014-2019 | 0.78 | -0.14~1.68 | 0.08 |
|  | 2019-2021 | -7.05 | -9.02~-4.38 | <0001 |
|  | AAPC(%) | -0.421 | -0.55~-0.3 | <0001 |
| **Male** | 1990-1997 | -0.38 | -0.76~0.51 | 0.21 |
|  | 1997-2004 | -1.5 | -2.75~-1.09 | 0.02 |
|  | 2004-2010 | 4.05 | 3.49~5.18 | <0001 |
|  | 2010-2019 | 1.93 | 1.61~2.25 | <0001 |
|  | 2019-2021 | -5.01 | -6.7~-2.44 | <0001 |
|  | AAPC(%) | 0.568 | 0.46~0.67 | <0001 |

APC, annual percentage change; AAPC, average annual percentage change; CI, confidence interval. Data are shown with 95% confidence intervals. Data source: Global Burden of Disease Study 2021
