# Supplementary material for: Long-term trends in the burden of leukemia subtypes in China from 1990 to 2021: a Joinpoint regression and age-period-cohort analysis based on GBD 2021
Source: Front Med (Lausanne). 2026 Jun 4;13:1826237. doi: 10.3389/fmed.2026.1826237 (PMC13275245; doi:10.3389/fmed.2026.1826237)
Supplement: Supplementary file 12 [file Table_6.docx]

**Table S6.** Age–period–cohort (APC) analysis of relative risks (RRs) for leukemia subtypes in China

| **Factor** | **AML** | | **CML** | | **ALL** | | **Factor** | **CLL** | |
| --- | --- | --- | --- | --- | --- | --- | --- | --- | --- |
|  | **RR(95%CI)** | **P** | **RR(95%CI)** | **P** | **RR(95%CI)** | **P** |  | **RR(95%CI)** | **P** |
| **age** |  |  |  |  |  |  | **age** |  |  |
| 0-4 | 2.07(1.98~2.17) | <0.001 | 0.71(0.64~0.78) | <0.001 | 5.37(5.04~5.72) | <0.001 | 20-24 | 0.32(0.31~0.34) | <0.001 |
| 5-9 | 0.6(0.58~0.63) | <0.001 | 0.42(0.38~0.46) | <0.001 | 1.46(1.38~1.55) | <0.001 | 25-29 | 0.29(0.28~0.3) | <0.001 |
| 10-14 | 0.52(0.5~0.54) | <0.001 | 0.38(0.35~0.41) | <0.001 | 1.14(1.09~1.2) | <0.001 | 30-34 | 0.4(0.38~0.42) | <0.001 |
| 15-19 | 0.57(0.55~0.59) | <0.001 | 0.4(0.38~0.43) | <0.001 | 1.16(1.11~1.21) | <0.001 | 35-39 | 0.48(0.47~0.5) | <0.001 |
| 20-24 | 0.41(0.4~0.42) | <0.001 | 0.53(0.5~0.56) | <0.001 | 0.62(0.59~0.64) | <0.001 | 40-44 | 0.68(0.66~0.69) | <0.001 |
| 25-29 | 0.4(0.39~0.41) | <0.001 | 0.55(0.52~0.58) | <0.001 | 0.54(0.53~0.56) | <0.001 | 45-49 | 0.71(0.7~0.73) | <0.001 |
| 30-34 | 0.45(0.44~0.46) | <0.001 | 0.64(0.61~0.67) | <0.001 | 0.55(0.54~0.57) | <0.001 | 50-54 | 0.99(0.98~1.01) | 0.4 |
| 35-39 | 0.58(0.57~0.59) | <0.001 | 0.83(0.8~0.86) | <0.001 | 0.68(0.67~0.69) | <0.001 | 55-59 | 1.27(1.25~1.29) | <0.001 |
| 40-44 | 0.64(0.63~0.65) | <0.001 | 0.81(0.78~0.84) | <0.001 | 0.66(0.65~0.67) | <0.001 | 60-64 | 1.7(1.67~1.72) | <0.001 |
| 45-49 | 0.66(0.65~0.67) | <0.001 | 0.94(0.91~0.97) | <0.001 | 0.65(0.64~0.66) | <0.001 | 65-69 | 1.77(1.74~1.81) | <0.001 |
| 50-54 | 0.82(0.81~0.83) | <0.001 | 1(0.97~1.03) | 0.94 | 0.7(0.69~0.71) | <0.001 | 70-74 | 2.16(2.11~2.21) | <0.001 |
| 55-59 | 1.02(1~1.04) | 0.04 | 1.19(1.15~1.23) | <0.001 | 0.88(0.87~0.9) | <0.001 | 75-79 | 2.49(2.42~2.57) | <0.001 |
| 60-64 | 1.22(1.19~1.24) | <0.001 | 1.31(1.25~1.37) | <0.001 | 1(0.98~1.03) | 0.69 | 80-84 | 1.62(1.56~1.69) | <0.001 |
| 65-69 | 1.57(1.53~1.61) | <0.001 | 1.51(1.44~1.59) | <0.001 | 1.1(1.06~1.13) | <0.001 | 85-89 | 2(1.91~2.1) | <0.001 |
| 70-74 | 1.91(1.86~1.97) | <0.001 | 2.16(2.04~2.3) | <0.001 | 1.35(1.3~1.4) | <0.001 | 90-94 | 1.6(1.5~1.7) | <0.001 |
| 75-79 | 2.16(2.09~2.23) | <0.001 | 2.3(2.15~2.47) | <0.001 | 1.45(1.38~1.51) | <0.001 | 95-99 | 1.08(0.96~1.22) | 0.2 |
| 80-84 | 2.24(2.15~2.33) | <0.001 | 2.19(2.02~2.37) | <0.001 | 1.28(1.22~1.34) | <0.001 |  |  |  |
| 85-89 | 2.96(2.83~3.1) | <0.001 | 2.68(2.44~2.96) | <0.001 | 1.23(1.16~1.3) | <0.001 |  |  |  |
| 90-94 | 2.81(2.65~2.98) | <0.001 | 2.43(2.13~2.76) | <0.001 | 1.13(1.04~1.22) | <0.001 |  |  |  |
| 95-99 | 1.23(1.06~1.43) | 0.01 | 1.13(0.81~1.57) | 0.48 | 0.75(0.63~0.9) | <0.001 |  |  |  |

(Continued on next page)

Table S6. Continued

| **Factor** | **AML** | | **CML** | | **ALL** | | **Factor** | **CLL** | |
| --- | --- | --- | --- | --- | --- | --- | --- | --- | --- |
|  | **RR(95%CI)** | **P** | **RR(95%CI)** | **P** | **RR(95%CI)** | **P** |  | **RR(95%CI)** | **P** |
| **period** |  |  |  |  |  |  | **period** |  |  |
| 1992-1996 | 0.99(0.97~1) | 0.04 | 1.17(1.14~1.2) | <0.001 | 1.11(1.09~1.13) | <0.001 | 1992-1996 | 0.65(0.64~0.66) | <0.001 |
| 1997-2001 | 1.02(1.01~1.03) | <0.001 | 1.13(1.11~1.16) | <0.001 | 1.04(1.03~1.05) | <0.001 | 1997-2001 | 0.75(0.74~0.76) | <0.001 |
| 2002-2006 | 1.06(1.05~1.06) | <0.001 | 1.06(1.05~1.08) | <0.001 | 0.95(0.95~0.96) | <0.001 | 2002-2006 | 0.92(0.92~0.93) | <0.001 |
| 2007-2011 | 0.99(0.98~1) | 0.01 | 0.94(0.93~0.95) | <0.001 | 0.99(0.98~1) | <0.001 | 2007-2011 | 1.12(1.11~1.13) | <0.001 |
| 2012-2016 | 0.94(0.93~0.95) | <0.001 | 0.85(0.83~0.87) | <0.001 | 0.99(0.97~1) | 0.01 | 2012-2016 | 1.32(1.31~1.34) | <0.001 |
| 2017-2021 | 1.01(0.99~1.02) | 0.22 | 0.89(0.86~0.92) | <0.001 | 0.93(0.91~0.94) | <0.001 | 2017-2021 | 1.5(1.47~1.53) | <0.001 |
| **cohort** |  |  |  |  |  |  | **cohort** |  |  |
| 1897-1901 | 1.81(0.97~3.37) | 0.06 | 1.77(0.47~6.65) | 0.4 | 0.84(0.35~1.98) | 0.69 | 1897-1901 | 2.23(1.23~4.06) | 0.01 |
| 1902-1906 | 1.57(1.33~1.85) | <0.001 | 1.72(1.22~2.44) | <0.001 | 0.92(0.72~1.18) | 0.51 | 1902-1906 | 2.06(1.7~2.5) | <0.001 |
| 1907-1911 | 1.44(1.3~1.59) | <0.001 | 1.55(1.26~1.91) | <0.001 | 0.91(0.79~1.04) | 0.17 | 1907-1911 | 1.84(1.64~2.06) | <0.001 |
| 1912-1916 | 1.39(1.28~1.51) | <0.001 | 1.51(1.27~1.8) | <0.001 | 0.93(0.83~1.04) | 0.2 | 1912-1916 | 1.63(1.48~1.79) | <0.001 |
| 1917-1921 | 1.34(1.25~1.44) | <0.001 | 1.52(1.3~1.77) | <0.001 | 1.01(0.91~1.11) | 0.86 | 1917-1921 | 1.32(1.21~1.43) | <0.001 |
| 1922-1926 | 1.31(1.22~1.4) | <0.001 | 1.51(1.32~1.74) | <0.001 | 1.06(0.97~1.16) | 0.23 | 1922-1926 | 1.14(1.06~1.23) | <0.001 |
| 1927-1931 | 1.32(1.24~1.4) | <0.001 | 1.46(1.29~1.66) | <0.001 | 1.07(0.98~1.16) | 0.12 | 1927-1931 | 1.05(0.98~1.12) | 0.17 |
| 1932-1936 | 1.32(1.24~1.39) | <0.001 | 1.4(1.24~1.57) | <0.001 | 1.07(0.99~1.16) | 0.08 | 1932-1936 | 0.97(0.92~1.03) | 0.31 |
| 1937-1941 | 1.3(1.23~1.36) | <0.001 | 1.3(1.17~1.45) | <0.001 | 1.07(1~1.14) | 0.07 | 1937-1941 | 0.93(0.89~0.98) | 0.01 |
| 1942-1946 | 1.23(1.17~1.29) | <0.001 | 1.21(1.1~1.34) | <0.001 | 1.06(0.99~1.13) | 0.07 | 1942-1946 | 0.92(0.88~0.96) | <0.001 |
| 1947-1951 | 1.18(1.14~1.23) | <0.001 | 1.28(1.17~1.39) | <0.001 | 1.08(1.02~1.14) | 0.01 | 1947-1951 | 0.92(0.89~0.96) | <0.001 |
| 1952-1956 | 1.13(1.09~1.18) | <0.001 | 1.29(1.19~1.39) | <0.001 | 1.04(0.99~1.1) | 0.09 | 1952-1956 | 0.9(0.88~0.93) | <0.001 |
| 1957-1961 | 1.14(1.1~1.17) | <0.001 | 1.23(1.15~1.31) | <0.001 | 0.97(0.93~1.01) | 0.16 | 1957-1961 | 0.9(0.88~0.92) | <0.001 |

(Continued on next page)

Table S6. Continued

| **Factor** | **AML** | | **CML** | | **ALL** | | **Factor** | **CLL** | |
| --- | --- | --- | --- | --- | --- | --- | --- | --- | --- |
|  | **RR(95%CI)** | **P** | **RR(95%CI)** | **P** | **RR(95%CI)** | **P** |  | **RR(95%CI)** | **P** |
| 1962-1966 | 1.05(1.02~1.08) | <0.001 | 1.19(1.12~1.26) | <0.001 | 0.92(0.89~0.95) | <0.001 | 1962-1966 | 0.86(0.85~0.88) | <0.001 |
| 1967-1971 | 1.01(0.98~1.03) | 0.52 | 1.17(1.11~1.23) | <0.001 | 0.91(0.89~0.94) | <0.001 | 1967-1971 | 0.87(0.86~0.88) | <0.001 |
| 1972-1976 | 0.97(0.95~0.99) | <0.001 | 1.04(1~1.09) | 0.05 | 0.84(0.82~0.86) | <0.001 | 1972-1976 | 0.8(0.79~0.81) | <0.001 |
| 1977-1981 | 1.05(1.03~1.06) | <0.001 | 1.01(0.97~1.05) | 0.69 | 0.83(0.82~0.84) | <0.001 | 1977-1981 | 0.74(0.73~0.75) | <0.001 |
| 1982-1986 | 1.07(1.05~1.08) | <0.001 | 1.04(1~1.07) | 0.03 | 0.88(0.87~0.89) | <0.001 | 1982-1986 | 0.64(0.62~0.65) | <0.001 |
| 1987-1991 | 0.99(0.97~1) | 0.04 | 1.02(0.99~1.05) | 0.25 | 0.94(0.94~0.95) | <0.001 | 1987-1991 | 0.63(0.61~0.64) | <0.001 |
| 1992-1996 | 0.96(0.95~0.98) | <0.001 | 0.9(0.87~0.93) | <0.001 | 0.87(0.87~0.88) | <0.001 | 1992-1996 | 0.64(0.61~0.66) | <0.001 |
| 1997-2001 | 0.88(0.86~0.89) | <0.001 | 0.78(0.74~0.82) | <0.001 | 0.91(0.89~0.92) | <0.001 | 1997-2001 | 0.65(0.62~0.69) | <0.001 |
| 2002-2006 | 0.66(0.65~0.68) | <0.001 | 0.59(0.55~0.62) | <0.001 | 0.99(0.97~1.02) | 0.59 |  |  |  |
| 2007-2011 | 0.48(0.46~0.49) | <0.001 | 0.4(0.37~0.44) | <0.001 | 1.18(1.15~1.21) | <0.001 |  |  |  |
| 2012-2016 | 0.35(0.34~0.37) | <0.001 | 0.27(0.24~0.3) | <0.001 | 1.42(1.37~1.47) | <0.001 |  |  |  |
| 2017-2021 | 0.22(0.21~0.23) | <0.001 | 0.14(0.11~0.17) | <0.001 | 1.59(1.52~1.65) | <0.001 |  |  |  |

Relative risks (RRs) and 95% confidence intervals (CIs) are presented for acute myeloid leukemia (AML), chronic myeloid leukemia (CML), acute lymphoblastic leukemia(ALL), and chronic lymphocytic leukemia(CLL) according to age group, calendar period, and birth cohort. Estimates were derived from APC modeling based on the Global Burden of Disease Study 2021.
