# Supplementary material for: Long-term trends in the burden of leukemia subtypes in China from 1990 to 2021: a Joinpoint regression and age-period-cohort analysis based on GBD 2021
Source: Front Med (Lausanne). 2026 Jun 4;13:1826237. doi: 10.3389/fmed.2026.1826237 (PMC13275245; doi:10.3389/fmed.2026.1826237)
Supplement: Supplementary file 13 [file Table_7.docx]

Table S7. Projected case numbers and age-standardized incidence rates (ASIRs) of leukemia subtypes in China, 2022–2046

| **Year** | **AML** | | **CML** | | **ALL** | | **CLL** | |
| --- | --- | --- | --- | --- | --- | --- | --- | --- |
|  | **Case number** | **ASIR** | **Case number** | **ASIR** | **Case number** | **ASIR** | **Case number** | **ASIR** |
| **2022** | 18759 | 1.01 | 4036 | 0.21 | 39102 | 3.44 | 29971 | 2.19 |
| **2023** | 19219 | 1.02 | 4136 | 0.21 | 38485 | 3.42 | 31022 | 2.22 |
| **2024** | 19703 | 1.02 | 4241 | 0.21 | 37896 | 3.39 | 32127 | 2.25 |
| **2025** | 20205 | 1.02 | 4347 | 0.21 | 37302 | 3.36 | 33262 | 2.28 |
| **2026** | 20722 | 1.02 | 4454 | 0.21 | 36698 | 3.33 | 34405 | 2.32 |
| **2027** | 21255 | 1.02 | 4560 | 0.21 | 36070 | 3.3 | 35532 | 2.35 |
| **2028** | 21807 | 1.02 | 4665 | 0.21 | 35467 | 3.27 | 36642 | 2.38 |
| **2029** | 22384 | 1.02 | 4772 | 0.21 | 34878 | 3.23 | 37751 | 2.41 |
| **2030** | 22984 | 1.02 | 4883 | 0.21 | 34305 | 3.2 | 38859 | 2.45 |
| **2031** | 23607 | 1.02 | 4998 | 0.21 | 33761 | 3.17 | 39970 | 2.48 |
| **2032** | 24259 | 1.03 | 5118 | 0.21 | 33249 | 3.13 | 41092 | 2.52 |
| **2033** | 24945 | 1.03 | 5247 | 0.21 | 32776 | 3.1 | 42221 | 2.55 |
| **2034** | 25670 | 1.03 | 5387 | 0.21 | 32340 | 3.07 | 43362 | 2.59 |
| **2035** | 26430 | 1.03 | 5536 | 0.21 | 31935 | 3.03 | 44528 | 2.62 |
| **2036** | 27223 | 1.03 | 5691 | 0.21 | 31567 | 3 | 45738 | 2.66 |
| **2037** | 28056 | 1.03 | 5851 | 0.21 | 31232 | 2.96 | 47018 | 2.7 |
| **2038** | 28948 | 1.03 | 6017 | 0.21 | 30999 | 2.93 | 48356 | 2.74 |
| **2039** | 29904 | 1.03 | 6194 | 0.21 | 30833 | 2.9 | 49769 | 2.78 |
| **2040** | 30912 | 1.03 | 6382 | 0.21 | 30723 | 2.87 | 51222 | 2.82 |
| **2041** | 31967 | 1.03 | 6575 | 0.21 | 30662 | 2.83 | 52684 | 2.86 |
| **2042** | 33069 | 1.03 | 6773 | 0.2 | 30638 | 2.8 | 54121 | 2.91 |
| **2043** | 34223 | 1.03 | 6975 | 0.2 | 30660 | 2.77 | 55526 | 2.95 |
| **2044** | 35440 | 1.03 | 7190 | 0.2 | 30726 | 2.74 | 56916 | 3 |
| **2045** | 36726 | 1.03 | 7416 | 0.2 | 30829 | 2.71 | 58312 | 3.05 |
| **2046** | 38098 | 1.03 | 7658 | 0.2 | 30968 | 2.68 | 59745 | 3.1 |

Data are presented as case number and ASIR (per 100,000 population) for acute myeloid leukemia (AML), chronic myeloid leukemia (CML), acute lymphoblastic leukemia (ALL), and chronic lymphocytic leukemia (CLL). Projections were generated using Bayesian age–period–cohort (BAPC) modeling based on data from the Global Burden of Disease Study 2021.
