# Supplementary material for: Long-term trends in the burden of leukemia subtypes in China from 1990 to 2021: a Joinpoint regression and age-period-cohort analysis based on GBD 2021
Source: Front Med (Lausanne). 2026 Jun 4;13:1826237. doi: 10.3389/fmed.2026.1826237 (PMC13275245; doi:10.3389/fmed.2026.1826237)
Supplement: Supplementary file 14 [file Table_8.docx]

Supplementary Table S8. Sex-specific DALYs attributable to four GBD-estimated risk factors for leukemia and its major subtypes in China, 1990–2021

| **Cause** | **Risk factors** | **Sex** | **1990** | | **2021** | | **1990-2021** | | |
| --- | --- | --- | --- | --- | --- | --- | --- | --- | --- |
|  |  |  | **DALYs cases (95% UI)** | **ASR of DALYs (95% UI)** | **DALYs cases (95% UI)** | **ASR of DALYs (95% UI)** | EAPCs (95% CI**）** | AAPC (95% CI**）** | ***P*** |
| **Leukemia** | High body-mass index | Female | 38171 (23964 to 55486) | 7.19 (4.52 to 10.42) | 60872 (35480 to 88857) | 6.43 (3.71 to 9.38) | -0.44 (-0.52,-0.35) | -0.33 (-0.55,-0.11) | 0.003 |
| **Leukemia** | High body-mass index | Male | 42874 (26624 to 62679) | 7.64 (4.77 to 11.24) | 83133 (50215 to 125169) | 9.21 (5.62 to 13.81) | 0.61 (0.56,0.67) | 0.62 (0.42,0.81) | <0.001 |
| **Leukemia** | Occupational exposure to benzene | Female | 8556 (2447 to 14743) | 1.4 (0.4 to 2.41) | 11265 (2949 to 20746) | 1.49 (0.39 to 2.73) | 0.03 (-0.08,0.14) | 0.20 (-0.04,0.43) | 0.099 |
| **Leukemia** | Occupational exposure to benzene | Male | 10415 (3012 to 18382) | 1.59 (0.46 to 2.81) | 11195 (3112 to 19859) | 1.41 (0.39 to 2.5) | -0.59 (-0.7,-0.49) | -0.37 (-0.68,-0.06) | 0.020 |
| **Leukemia** | Occupational exposure to formaldehyde | Female | 4268 (2830 to 5971) | 0.7 (0.47 to 0.97) | 4719 (2868 to 6966) | 0.63 (0.37 to 0.94) | -0.5 (-0.68,-0.33) | -0.36 (-0.60,-0.11) | 0.004 |
| **Leukemia** | Occupational exposure to formaldehyde | Male | 4989 (3125 to 7274) | 0.76 (0.48 to 1.12) | 6242 (3789 to 9013) | 0.79 (0.47 to 1.15) | -0.05 (-0.16,0.05) | 0.14 (-0.22,0.50) | 0.444 |
| **Leukemia** | Smoking | Female | 4158 (1483 to 7457) | 0.96 (0.33 to 1.75) | 6867 (2105 to 13416) | 0.61 (0.19 to 1.2) | -1.6 (-1.69,-1.51) | -1.41 (-1.71,-1.11) | <0.001 |
| **Leukemia** | Smoking | Male | 86993 (35706 to 143981) | 20.13 (8.18 to 33.25) | 180939 (68754 to 320856) | 17.37 (6.6 to 30.48) | -0.32 (-0.39,-0.24) | -0.48 (-0.61,-0.36) | <0.001 |
| **AML** | High body-mass index | Female | 8172 (3928 to 14025) | 1.57 (0.76 to 2.69) | 16614 (9617 to 28081) | 1.74 (1 to 2.96) | 0.07 (-0.14,0.28) | 0.33 (0.15,0.51) | <0.001 |
| **AML** | High body-mass index | Male | 9030 (3742 to 15422) | 1.64 (0.68 to 2.84) | 20192 (10036 to 32486) | 2.22 (1.12 to 3.54) | 0.89 (0.72,1.05) | 0.99 (0.89,1.10) | <0.001 |
| **AML** | Occupational exposure to benzene | Female | 1720 (402 to 3594) | 0.28 (0.07 to 0.58) | 3050 (841 to 5746) | 0.4 (0.11 to 0.75) | 0.85 (0.57,1.14) | 1.12 (0.75,1.49) | <0.001 |
| **AML** | Occupational exposure to benzene | Male | 2143 (521 to 4317) | 0.33 (0.08 to 0.66) | 2650 (738 to 4842) | 0.33 (0.09 to 0.61) | -0.21 (-0.46,0.05) | 0.01 (-0.15,0.18) | 0.872 |
| **AML** | Occupational exposure to formaldehyde | Female | 855 (412 to 1492) | 0.14 (0.07 to 0.25) | 1259 (737 to 2137) | 0.17 (0.1 to 0.28) | 0.27 (-0.09,0.63) | 0.52 (0.20,0.86) | 0.002 |

(Continued on next page)

Supplementary Table S8. Continued

| **Cause** | **Risk factors** | **Sex** | **1990** | | **2021** | | **1990-2021** | | |
| --- | --- | --- | --- | --- | --- | --- | --- | --- | --- |
|  |  |  | **DALYs cases (95% UI)** | **ASR of DALYs (95% UI)** | **DALYs cases (95% UI)** | **ASR of DALYs (95% UI)** | EAPCs (95% CI**）** | AAPC (95% CI**）** | ***P*** |

| **AML** | Occupational exposure to formaldehyde | Male | 1037 (410 to 1710) | 0.16 (0.06 to 0.26) | 1485 (761 to 2356) | 0.19 (0.09 to 0.29) | 0.32 (0.05,0.59) | 0.50 (0.32,0.67) | <0.001 |
| --- | --- | --- | --- | --- | --- | --- | --- | --- | --- |
| **AML** | Smoking | Female | 972 (318 to 1969) | 0.23 (0.07 to 0.46) | 1882 (563 to 4033) | 0.17 (0.05 to 0.36) | -1.41 (-1.61,-1.2) | -0.92 (-1.04,-0.80) | <0.001 |
| **AML** | Smoking | Male | 19310 (6213 to 35384) | 4.58 (1.51 to 8.21) | 44340 (15603 to 83220) | 4.26 (1.47 to 8.01) | -0.28 (-0.37,-0.2) | -0.24 (-0.44,-0.05) | 0.015 |
| **CML** | High body-mass index | Female | 2904 (1238 to 5376) | 0.55 (0.24 to 1.02) | 2010 (1117 to 3576) | 0.21 (0.12 to 0.36) | -3.8 (-4.1,-3.49) | -3.05 (-3.41,-2.69) | <0.001 |
| **CML** | High body-mass index | Male | 2806 (578 to 4976) | 0.5 (0.1 to 0.88) | 2620 (717 to 5268) | 0.29 (0.08 to 0.58) | -2.36 (-2.64,-2.08) | -1.73 (-2.11,-1.34) | <0.001 |
| **CML** | Occupational exposure to benzene | Female | 650 (156 to 1261) | 0.11 (0.03 to 0.21) | 357 (89 to 760) | 0.05 (0.01 to 0.1) | -3.68 (-4.05,-3.31) | -2.70 (-3.07,-2.32) | <0.001 |
| **CML** | Occupational exposure to benzene | Male | 691 (106 to 1432) | 0.11 (0.02 to 0.22) | 358 (80 to 784) | 0.04 (0.01 to 0.1) | -3.78 (-4.17,-3.4) | -2.83 (-3.42,-2.24) | <0.001 |
| **CML** | Occupational exposure to formaldehyde | Female | 325 (139 to 584) | 0.05 (0.02 to 0.1) | 146 (84 to 252) | 0.02 (0.01 to 0.03) | -4.28 (-4.69,-3.87) | -3.32 (-3.67,-2.96) | <0.001 |
| **CML** | Occupational exposure to formaldehyde | Male | 338 (76 to 572) | 0.05 (0.01 to 0.09) | 203 (52 to 386) | 0.03 (0.01 to 0.05) | -3.28 (-3.67,-2.9) | -2.34 (-2.79,-1.89) | <0.001 |
| **CML** | Smoking | Female | 312 (96 to 643) | 0.07 (0.02 to 0.15) | 236 (72 to 518) | 0.02 (0.01 to 0.05) | -4.57 (-4.9,-4.23) | -3.90 (-4.20,-3.61) | <0.001 |
| **CML** | Smoking | Male | 5504 (950 to 10735) | 1.27 (0.22 to 2.46) | 5545 (1139 to 12867) | 0.54 (0.11 to 1.25) | -2.98 (-3.18,-2.77) | -2.76 (-3.08,-2.43) | <0.001 |
| **ALL** | High body-mass index | Female | 14779 (8172 to 23599) | 2.68 (1.47 to 4.27) | 19539 (7321 to 30700) | 2.16 (0.83 to 3.33) | -0.68 (-0.74,-0.62) | -0.69 (-0.84,-0.55) | <0.001 |
| **ALL** | High body-mass index | Male | 15632 (8022 to 25082) | 2.63 (1.35 to 4.29) | 27831 (12139 to 44497) | 3.19 (1.4 to 5.01) | 0.7 (0.6,0.8) | 0.61 (0.45,0.78) | <0.001 |

(Continued on next page)

Supplementary Table S8. Continued

| **Cause** | **Risk factors** | **Sex** | **1990** | | **2021** | | **1990-2021** | | |
| --- | --- | --- | --- | --- | --- | --- | --- | --- | --- |
|  |  |  | **DALYs cases (95% UI)** | **ASR of DALYs (95% UI)** | **DALYs cases (95% UI)** | **ASR of DALYs (95% UI)** | EAPCs (95% CI**）** | AAPC (95% CI**）** | ***P*** |

| **ALL** | Occupational exposure to benzene | Female | 3833 (1057 to 7215) | 0.61 (0.17 to 1.16) | 4141 (969 to 7955) | 0.58 (0.13 to 1.11) | -0.3 (-0.37,-0.23) | -0.21 (-0.51,0.09) | 0.172 |
| --- | --- | --- | --- | --- | --- | --- | --- | --- | --- |
| **ALL** | Occupational exposure to benzene | Male | 4415 (1084 to 8122) | 0.66 (0.16 to 1.21) | 4350 (1142 to 7920) | 0.57 (0.15 to 1.04) | -0.67 (-0.78,-0.56) | -0.44 (-0.72,-0.15) | 0.003 |
| **ALL** | Occupational exposure to formaldehyde | Female | 1916 (1008 to 2920) | 0.31 (0.16 to 0.47) | 1749 (691 to 2795) | 0.24 (0.1 to 0.4) | -0.81 (-0.91,-0.72) | -0.76 (-1.07,-0.44) | <0.001 |
| **ALL** | Occupational exposure to formaldehyde | Male | 2102 (1140 to 3305) | 0.31 (0.17 to 0.49) | 2413 (1033 to 3692) | 0.32 (0.13 to 0.48) | -0.13 (-0.22,-0.03) | 0.06 (-0.23,0.35) | 0.685 |
| **ALL** | Smoking | Female | 1333 (408 to 2696) | 0.3 (0.09 to 0.6) | 1986 (473 to 4190) | 0.18 (0.04 to 0.37) | -1.57 (-1.64,-1.5) | -1.65 (-1.88,-1.42) | <0.001 |
| **ALL** | Smoking | Male | 26180 (9732 to 46321) | 5.73 (2.15 to 10.01) | 52103 (16574 to 98862) | 4.95 (1.59 to 9.4) | -0.07 (-0.24,0.09) | -0.49 (-0.56,-0.42) | <0.001 |
| **CLL** | High body-mass index | Female | 3923 (1682 to 6941) | 0.78 (0.35 to 1.37) | 8730 (3326 to 13743) | 0.88 (0.34 to 1.38) | 0.37 (0.28,0.47) | 0.43 (0.19,0.67) | <0.001 |
| **CLL** | High body-mass index | Male | 5035 (2203 to 8251) | 0.98 (0.44 to 1.6) | 13567 (6586 to 22729) | 1.44 (0.7 to 2.38) | 1.29 (1.21,1.36) | 1.24 (1.11,1.38) | <0.001 |
| **CLL** | Occupational exposure to benzene | Female | 709 (171 to 1529) | 0.12 (0.03 to 0.26) | 1319 (284 to 2597) | 0.16 (0.03 to 0.31) | 0.8 (0.69,0.92) | 0.91 (0.49,1.32) | <0.001 |
| **CLL** | Occupational exposure to benzene | Male | 967 (215 to 1788) | 0.16 (0.03 to 0.29) | 1476 (379 to 2790) | 0.17 (0.04 to 0.33) | 0.28 (0.14,0.43) | 0.34 (0.10,0.57) | 0.005 |
| **CLL** | Occupational exposure to formaldehyde | Female | 353 (140 to 651) | 0.06 (0.02 to 0.11) | 558 (215 to 895) | 0.07 (0.03 to 0.11) | 0.31 (0.14,0.47) | 0.40 (-0.05,0.84) | 0.079 |
| **CLL** | Occupational exposure to formaldehyde | Male | 460 (190 to 704) | 0.07 (0.03 to 0.11) | 823 (412 to 1375) | 0.1 (0.05 to 0.16) | 0.84 (0.69,0.99) | 0.86 (0.63,1.08) | <0.001 |
| **CLL** | Smoking | Female | 544 (168 to 1067) | 0.13 (0.04 to 0.26) | 1147 (311 to 2343) | 0.1 (0.03 to 0.21) | -0.99 (-1.12,-0.87) | -0.79 (-1.05,-0.52) | <0.001 |

(Continued on next page)

Supplementary Table S8. Continued

| **Cause** | **Risk factors** | **Sex** | **1990** | | **2021** | | **1990-2021** | | |
| --- | --- | --- | --- | --- | --- | --- | --- | --- | --- |
|  |  |  | **DALYs cases (95% UI)** | **ASR of DALYs (95% UI)** | **DALYs cases (95% UI)** | **ASR of DALYs (95% UI)** | EAPCs (95% CI**）** | AAPC (95% CI**）** | ***P*** |

| **CLL** | Smoking | Male | 13319 (3729 to 23890) | 3.26 (0.88 to 5.84) | 35773 (10376 to 73700) | 3.47 (1.01 to 7.04) | 0.27 (0.21,0.33) | 0.18 (0.02,0.34) | 0.031 |
| --- | --- | --- | --- | --- | --- | --- | --- | --- | --- |

DALYs and age-standardized DALY rates are presented with 95% UIs. EAPCs and AAPCs are presented with 95% CIs. P values refer to AAPCs. DALYs, disability-adjusted life years; ASR, age-standardized rate; EAPC, estimated annual percentage change; AAPC, average annual percentage change; UI, uncertainty interval; CI, confidence interval; AML, acute myeloid leukemia; CML, chronic myeloid leukemia; ALL, acute lymphoblastic leukemia; CLL, chronic lymphocytic leukemia.
